# A Mendelian randomization study of the effect of mental disorders on cardiovascular disease

**DOI:** 10.3389/fcvm.2024.1329463

**Published:** 2024-06-03

**Authors:** Yunfeng Yu, Xinyu Yang, Jingyi Wu, Gang Hu, Siyang Bai, Rong Yu

**Affiliations:** ^1^ School of Traditional Chinese Medicine, Hunan University of Chinese Medicine, Changsha, Hunan, China; ^2^ The First Hospital of Hunan University of Chinese Medicine, Changsha, Hunan, China; ^3^ The Third School of Clinical Medicine, Zhejiang Chinese Medical University, Hangzhou, Zhejiang, China

**Keywords:** mental disorders, cardiovascular disease, risk factor, Mendelian randomization, depression, anxiety disorder, autism spectrum disorder

## Abstract

**Objective:**

The effect of mental disorders (MD) on cardiovascular disease (CVD) remains controversial, and this study aims to analyze the causal relationship between eight MD and CVD by Mendelian randomization (MR).

**Methods:**

Single nucleotide polymorphisms of attention-deficit/hyperactivity disorder (ADHD), anorexia nervosa (AN), anxiety disorder (ANX), autism spectrum disorder (ASD), bipolar disorder (BD), depression, obsessive-compulsive disorder (OCD), schizophrenia (SCZ), and CVD were obtained from UK Biobank and FinnGen. Exposure-outcome causality was tested using inverse variance weighted (IVW), MR-Egger, and weighted median. Horizontal pleiotropy and heterogeneity were assessed by MR-Egger intercept and Cochran's Q, respectively, while stability of results was assessed by leave-one-out sensitivity analysis.

**Results:**

MR analysis showed that ANX (IVW [odds ratio (OR) 1.11, 95% confidence intervals (CI) 1.07–1.15, *p* < 0.001]; MR-Egger [OR 1.03, 95% CI 0.92–1.14, *p* = 0.652]; weighted median [OR 1.09, 95% CI 1.03–1.14, *p* = 0.001]), ASD (IVW [OR 1.05, 95% CI 1.00–1.09, *p* = 0.039]; MR-Egger [OR 0.95, 95% CI 0.84–1.07, *p* = 0.411]; weighted median [OR 1.01, 95% CI 0.96–1.06, *p* = 0.805]), depression (IVW [OR 1.15, 95% CI 1.10–1.19, *p* < 0.001]; MR-Egger [OR 1.10, 95% CI 0.96–1.26, *p* = 0.169]; weighted median [OR 1.13, 95% CI 1.08–1.19, *p* < 0.001]) were significantly associated with increased risk of CVD, whereas ADHD, AN, BD, OCD, and SCZ were not significantly associated with CVD (*p* > 0.05). Intercept analysis showed no horizontal pleiotropy (*p* > 0.05). Cochran's Q showed no heterogeneity except for BD (*p* = 0.035). Sensitivity analysis suggested that these results were robust.

**Conclusions:**

ANX, ASD, and depression are associated with an increased risk of CVD, whereas AN, ADHD, BD, OCD, and SCZ are not causally associated with CVD. Active prevention and treatment of ANX, ASD, and depression may help reduce the risk of CVD.

## 1 Introduction

Cardiovascular disease (CVD), a circulatory system disease involving lesions of the heart and blood vessels (1, 2), is the leading cause of disability and premature death in adults (3). Epidemiological research showed that in 2019, about 523 million people worldwide had CVD, about 18.6 million people died from CVD, and 23.6 million people were expected to die from CVD annually by 2030 (4, 5). CVD remains a significant threat to human health, and controlling associated risk factors is an essential means of preventing and treating CVD (6). Smoking, obesity, and unhealthy diet are considered to be common risk factors for CVD (7, 8), and the control of these risk factors effectively reduces the risk of CVD (9). In recent years, with the development of the biopsychosocial model, people have become concerned about the influence of psychosocial factors on disease, and there has been growing evidence that mental disorders (MD) may be potential risk factors for CVD (10, 11).

MD are chronic heterogeneous diseases characterized by abnormal mental or behavioral patterns (12, 13). They include attention-deficit/hyperactivity disorder (ADHD), anorexia nervosa (AN), anxiety disorder (ANX), autism spectrum disorder (ASD), bipolar disorder (BD), depression, obsessive-compulsive disorder (OCD), schizophrenia (SCZ), and so on. As a global health problem, MD are characterized by high prevalence and high cost, which brings a huge burden to individuals, families, and society (14). Previous studies have shown that depression increases the risk of adverse cardiovascular events such as acute myocardial infarction, atrial fibrillation, heart failure, and stroke (15, 16). However, the effects of other MD on CVD remain controversial. Given that ADHD, AN, ANX, ASD, BD, depression, OCD, and SCZ are among the most prevalent and debilitating mental health problems, this study employed them as exposure factors to assess the causality between MD and CVD using Mendelian randomization (MR).

## 2 Materials and methods

### 2.1 Study design

**Figure 1:**
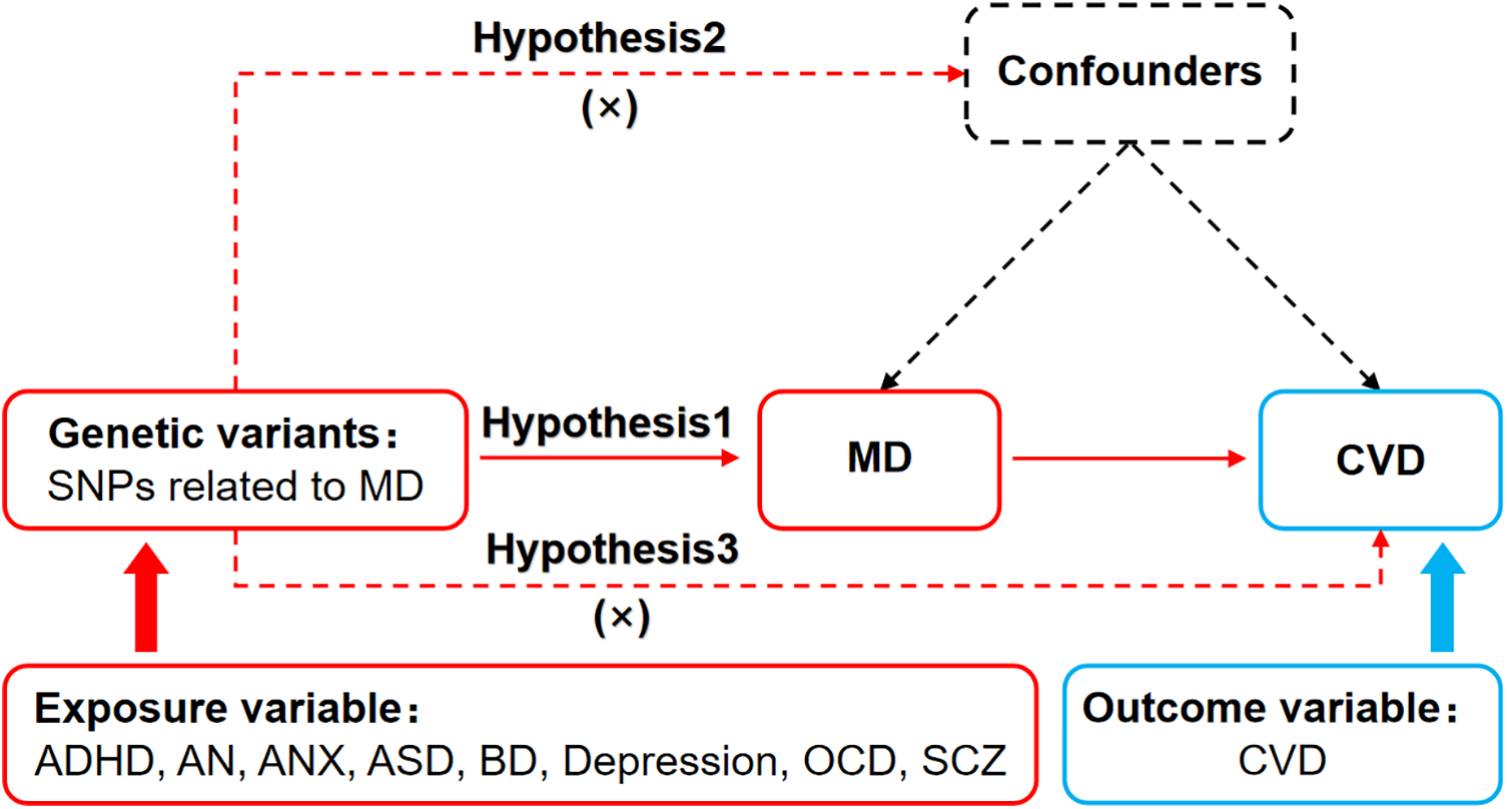
MR design for causal analysis of MD and CVD. MD, mental disorders; CVD, cardiovascular diseases.

Mendelian Randomization relied on three basic assumptions: (1) The association assumption: Single nucleotide polymorphisms (SNPs) were strongly associated with exposure factors. (2) The independence assumption: SNPs were independent of confounding factors. (3) The exclusivity assumption: SNPs could not act on outcome variables through pathways other than exposure factors. The MR design for MD and CVD is shown in Figure 1.

### 2.2 Data sources

With the exclusion of horizontal pleiotropy, this study selected the latest and largest sample size data for each exposure and outcome. The data for ADHD (dataset number: ieu-a-1183), AN (dataset number: ieu-a-1186), ASD (dataset number: ieu-a-1185), and BD (dataset number: ieu-b-41) were from UK Biobank (www.nealelab.is/uk-biobank), and the data for ANX (dataset number: finn-b-F5_ALLANXIOUS), depression (dataset number: finn-b-F5_DEPRESSIO), OCD (dataset number: finn-b-F5_OCD), SCZ (dataset number: finn-b-F5_SCHZPHR), and CVD (dataset number: finn-b-FG_CVD) were from FinnGen (www.finngen.fi/fi). All the data were obtained from publicly available databases, thus eliminating the need for additional ethical approval.

### 2.3 Selection of genetic instrument variables

The genome-wide association studies (GWAS) database contained data from eight exposure factors totaling 1990,114 individuals of European ancestry. First, SNPs strongly associated with the exposure factors were screened according to *p* < 5 × 10^–8^ to fulfill assumption 1. Second, independent SNPs were further screened according to *R*^2^ < 0.001 and kb = 10,000 to avoid potential bias due to linkage disequilibrium. Third, the F-value of each SNP was calculated, and SNPs with F ≤ 10 were excluded. F-value was calculated publicly as $F=[R^{2}/(1-R^{2})]\;*\;[(N-K-1)/k]$. $R^{2}=2\;*\;(1-\mathrm{MAF})\;*\;\mathrm{MAF}\;*\;\beta^{2}$. *R*^2^: the cumulative explained variance of the selected IVs on exposure; MAF: the minor allele frequency; β: estimated effect of SNP; *N*: sample size of the GWAS. Fourth, referring to PhenoScanner (www.phenoscanner.medschl.cam.ac.uk) and related literature to remove SNPs potentially associated with CVD to fulfill assumption 2. Finally, mismatched SNPs were excluded based on the effect allele frequency while harmonizing the allelic orientation. The remaining SNPs were used to perform MR analysis.

### 2.4 Data analysis

The study followed the STROBE-MR guidelines (17). The MR analysis was performed using the “TwoSampleMR (0.5.7)” package of the R 4.3.1, and inverse variance weighted (IVW), MR-Egger, and weighted median were used to assess causation. IVW is the primary method (18), which achieves unbiased causal estimation without horizontal pleiotropy and is the most informative. MR-Egger and weighted median are complementary methods for MR analysis. MR-Egger provides valid causal estimation in some cases where pleiotropy exists, and weighted median has a lower sensitivity to outliers and measurement errors.

MR results were corrected by using the MR-Pleiotropy RESidual Sum and Outlier method (MR-PRESSO), and MR analysis was re-executed after removing outlier (*p* < 1) SNPs. Horizontal pleiotropy was assessed using MR-Egger intercept analysis, with *p* ≥ 0.05 suggesting the absence of horizontal pleiotropy to fulfill assumption 3. Heterogeneity was assessed using Cochran's Q, with *p* ≥ 0.05 suggesting the absence of heterogeneity. Leave-one-out sensitivity analysis was used to assess the robustness of the results and to clarify the presence of individual SNPs that significantly affected the pooled results.

## 3 Results

### 3.1 GWAS data on exposure and outcomes

Details of the GWAS included in this study are shown in Table 1, with data on MD from UK Biobank and FinnGen, including 1990,114 Europeans. Data on CVD were from the FinnGen, including 377,277 Europeans. After excluding the effects of linkage disequilibrium and confounders, the included SNPs are shown in Supplementary Table S1. Subsequently, mismatched SNPs were excluded when harmonizing the allelic orientations of the exposure-SNPs and the outcome-SNPs. And outlier SNPs were excluded in the MR-PRESSO correction. Finnaly, SNPs for causal analysis of MD and CVD are shown in Supplementary Table S2.

**Table 1:** Details of the GWAS studies included in the mendelian randomization.

Year	Trait	Population	Sample size	Web source
2023	CVD	European	377,277	www.finngen.fi/en
2017	ADHD	European	55,374	www.nealelab.is/uk-biobank
2017	AN	European	14,477	www.nealelab.is/uk-biobank
2023	ANX	European	362,239	www.finngen.fi/en
2017	ASD	European	46,351	www.nealelab.is/uk-biobank
2019	BD	European	51,710	www.nealelab.is/uk-biobank
2023	Depression	European	372,472	www.finngen.fi/en
2023	OCD	European	339,539	www.finngen.fi/en
2023	SCZ	European	370,675	www.finngen.fi/en

CVD, cardiovascular diseases; ADHD, attention deficit hyperactivity disorder; AN, anorexia nervosa; ANX, anxiety disorder; ASD, autism spectrum disorder; BD, bipolar disorder; OCD, obsessive compulsive disorder; SCZ, schizophrenia.

### 3.2 Mr analysis results

**Figure 2:**
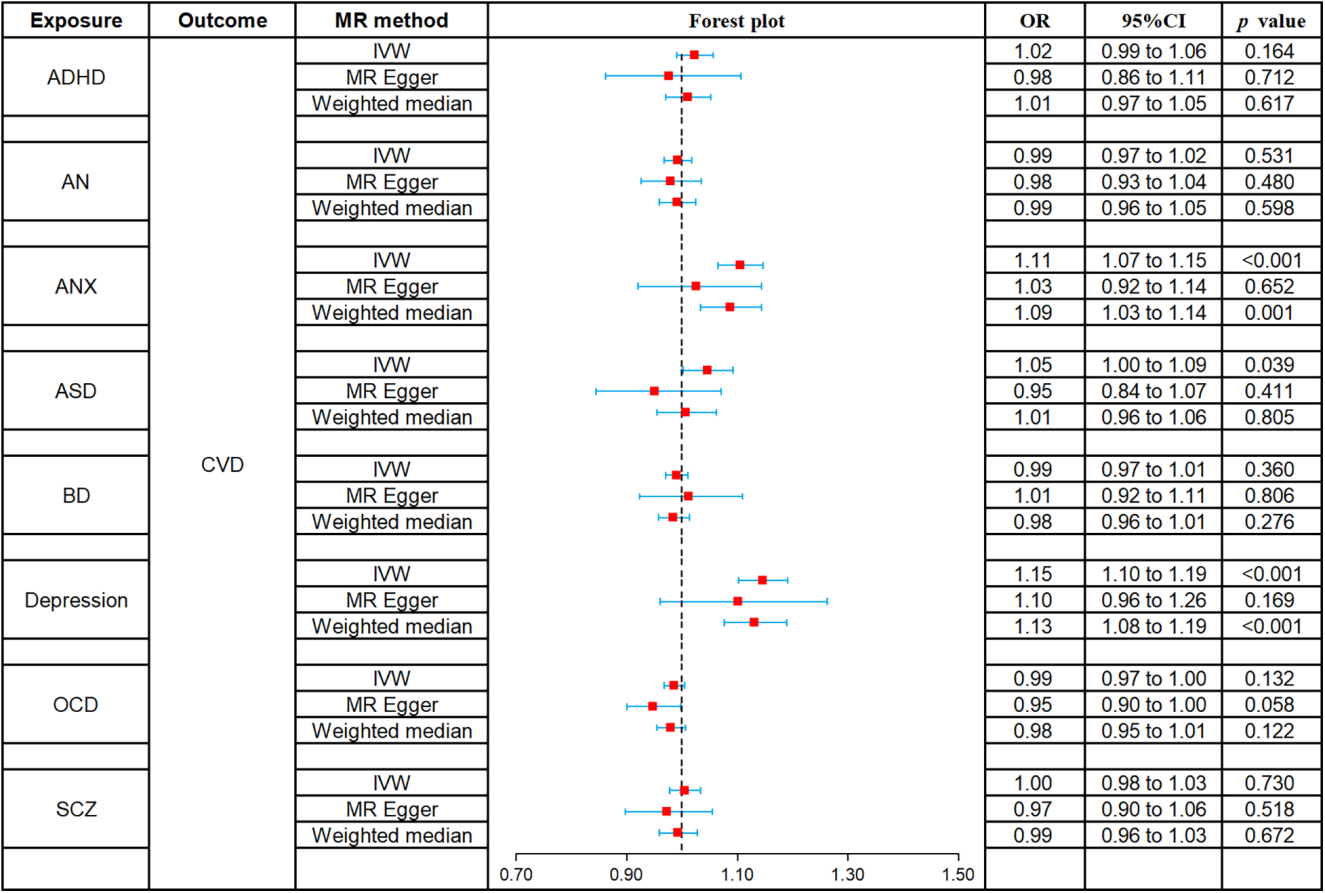
Forest plot of MR analysis on the causal relationship between MD and CVD. (**A**) ADHD on CVD; (**B**) AN on CVD; (**C**) ANX on CVD; (**D**) ASD on CVD; (**E**) BD on CVD; (**F**) Depression on CVD; (**G**) OCD on CVD; (**H**) SCZ on CVD. MD, mental disorders; CVD, cardiovascular diseases; ADHD, attention deficit hyperactivity disorder; AN, anorexia nervosa; ANX, anxiety disorder; ASD, autism spectrum disorder; BD, bipolar disorder; OCD, obsessive compulsive disorder; SCZ, schizophrenia.

**Figure 3:**
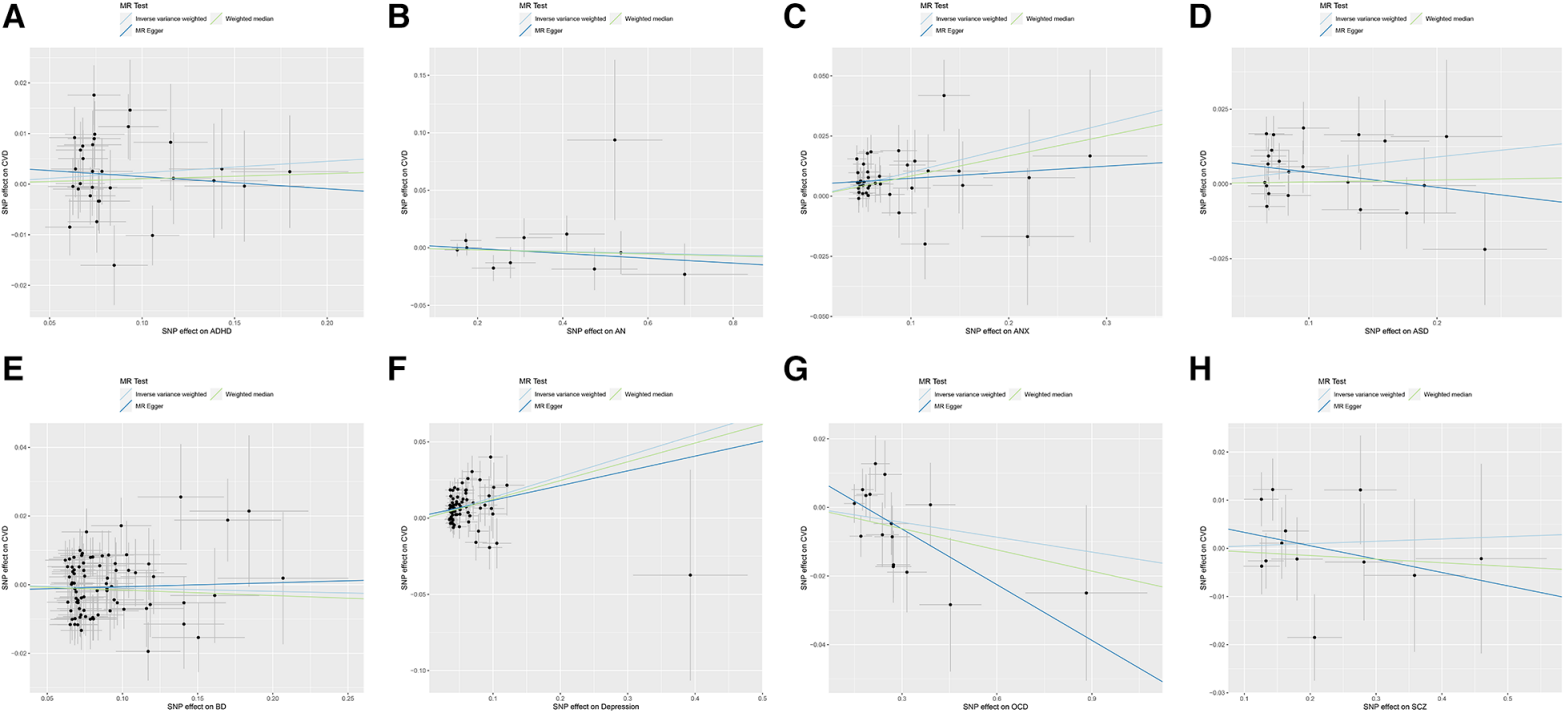
Scatter plot of MR analysis on the causal relationship between MD and CVD. (**A**) ADHD on CVD; (**B**) AN on CVD; (**C**) ANX on CVD; (**D**) ASD on CVD; (**E**) BD on CVD; (**F**) Depression on CVD; (**G**) OCD on CVD; (**H**) SCZ on CVD. MD, mental disorders; CVD, cardiovascular diseases; ADHD, attention deficit hyperactivity disorder; AN, anorexia nervosa; ANX, anxiety disorder; ASD, autism spectrum disorder; BD, bipolar disorder; OCD, obsessive compulsive disorder; SCZ, schizophrenia.

The causal relationships of ADHD, AN, ANX, ASD, BD, depression, OCD, and SCZ with CVD were analyzed using MR. The forest plot of the MR analysis is shown in Figure 2, and the effect estimates for each SNP are shown in Figure 3. MR-Egger intercept analysis is shown in Supplementary Table S3, heterogeneity analysis is shown in Supplementary Figure S1; Supplementary Table S4, and sensitivity analysis is shown in Supplementary Figure S2.

#### 3.2.1 ADHD

All three methods of analysis showed no significant causal relationship between ADHD and CVD: IVW (OR 1.02, 95% CI 0.99–1.06, *p* = 0.164), MR-Egger (OR 0.98, 95% CI 0.86–1.11, *p* = 0.712), and weighted median (OR 1.01, 95% CI 0.97–1.05, *p* = 0.617). Intercept analysis showed no horizontal pleiotropy (*p* = 0.460), heterogeneity test showed no significant heterogeneity (*p* = 0.177), and sensitivity analysis suggested the results were robust.

#### 3.2.2 AN

All three methods of analysis showed no significant causal relationship between AN and CVD: IVW (OR 0.99, 95% CI 0.97–1.02, *p* = 0.531), MR-Egger (OR 0.98, 95% CI 0.93–1.04, *p* = 0.480), and weighted median (OR 0.99, 95% CI 0.96–1.05, *p* = 0.598). Intercept analysis showed no horizontal pleiotropy (*p* = 0.622), heterogeneity test showed no significant heterogeneity (*p* = 0.582), and sensitivity analysis suggested the results were robust.

#### 3.2.3 ANX

IVW (OR 1.11, 95% CI 1.07–1.15, *p* < 0.001) and weighted median (OR 1.09, 95% CI 1.03–1.14, *p* = 0.001) showed that ANX was associated with increased risk of CVD, whereas no such causal relationship was observed for MR-Egger (OR 1.03, 95% CI 0.92–1.14, *p* = 0.652). Intercept analysis showed no horizontal pleiotropy (*p* = 0.159), heterogeneity test showed no significant heterogeneity (*p* = 0.731), and sensitivity analysis suggested the results were robust.

#### 3.2.4 ASD

IVW (OR 1.05, 95% CI 1.00–1.09, *p* = 0.039) showed that ASD was associated with increased risk of CVD, while MR-Egger (OR 0.95, 95% CI 0.84–1.07, *p* = 0.411) and weighted median (OR 1.01, 95% CI 0.96–1.06, *p* = 0.805) did not observe this causal relationship. Intercept analysis showed no horizontal pleiotropy (*p* = 0.106), heterogeneity test showed no significant heterogeneity (*p* = 0.059), and sensitivity analysis suggested the results were robust.

#### 3.2.5 BD

All three methods of analysis showed no significant causal relationship between BD and CVD: IVW (OR 0.99, 95% CI 0.97–1.01, *p* = 0.360), MR-Egger (OR 1.01, 95% CI 0.92–1.11, *p* = 0.806), and weighted median (OR 0.98, 95% CI 0.96–1.01, *p* = 0.276). Intercept analysis showed no horizontal pleiotropy (*p* = 0.643), heterogeneity test showed heterogeneity (*p* = 0.035), and sensitivity analysis suggested the results were robust.

#### 3.2.6 Depression

Both IVW (OR 1.15, 95% CI 1.10–1.19, *p* < 0.001) and weighted median (OR 1.13, 95% CI 1.08–1.19, *p* < 0.001) showed that BD was associated with increased risk of CVD, while MR-Egger (OR 1.10, 95% CI 0.96–1.26, *p* = 0.169) did not observe such a causal relationship. Intercept analysis showed no horizontal pleiotropy (*p* = 0.554), heterogeneity test showed no significant heterogeneity (*p* = 0.063), and sensitivity analysis suggested the results were robust.

#### 3.2.7 OCD

All three methods of analysis showed no significant causal relationship between OCD and CVD: IVW (OR 0.99, 95% CI 0.97–1.00, *p* = 0.132), MR-Egger (OR 0.95, 95% CI 0.90–1.00, *p* = 0.058), and weighted median (OR 0.98, 95% CI 0.95–1.01, *p* = 0.122). Intercept analysis showed no horizontal pleiotropy (*p* = 0.130), heterogeneity test showed no significant heterogeneity (*p* = 0.305). Sensitivity analysis suggested that rs80216287 was a significant SNP affecting the combined outcome, but detailed information about it could not be retrieved from the database. Hence, there is no justification for its exclusion.

#### 3.2.8 SCZ

All three methods of analysis showed no significant causal relationship between SCZ and CVD: IVW (OR 1.00, 95% CI 0.98–1.03, *p* = 0.730), MR-Egger (OR 0.97, 95% CI 0.90–1.06, *p* = 0.518), and weighted median (OR 0.99, 95% CI 0.96–1.03, *p* = 0.672). Intercept analysis showed no horizontal pleiotropy (*p* = 0.420), heterogeneity test showed no significant heterogeneity (*p* = 0.278), and sensitivity analysis suggested the results were robust.

## 4 Discussion

CVD, a group of diseases that involve pathological changes in the heart and systemic vasculature (19), is one of the leading causes of increased mortality worldwide (20). With the development of the biopsychosocial model, more and more researchers have found a close link between psychological factors and CVD (21). Studies have shown that MD may be an independent risk factor for CVD (22). They increase the morbidity and mortality of CVD patients and reduce the quality of survival in CVD patients (22, 23).

Previous MR analyses have found that ANX is linked to an elevated risk of coronary heart disease, myocardial infarction, and heart failure (24); ASD is associated with an increased risk of atrial fibrillation (25); depression is correlated with an increased risk of coronary heart disease and myocardial infarction (26); and SCZ is linked to an increased risk of heart failure (27). While these studies delve into the impact of specific types of MD on distinct cardiovascular events, they lack a comprehensive assessment of the different types of MD and the overall risk of CVD. In this study, we conducted a comprehensive assessment of different MD types on the overall risk of CVD, using MD such as ADHD, AN, ANX, ASD, BD, depression, OCD, and SCZ as exposure factors and CVD as the outcome variable. The MR analysis revealed a significant causal relationship of depression, ANX, and ASD with CVD, whereas no significant causal relationship of ADHD, AN, BD, OCD, and SCZ with CVD. These results were free of horizontal pleiotropy and heterogeneity and were confirmed to be robust through sensitivity analysis. This study provides additional support for the association of ANX, ASD, and depression with cardiovascular risk compared to previous findings, while refuting the notion of SCZ significantly affecting the overall CVD risk. It means that SCZ may only elevate the risk of heart failure but has a non-significant effect on the overall cardiovascular risk. In addition, this study points out that ADHD, AN, BD, and SCZ are not associated with the overall risk of CVD.

A previous cohort study has shown that depression increases the risk of CVD. A cohort study of Chinese older adults showed a 5% increased risk of CVD in those with depression compared with those without depression (28). A study in Hong Kong has shown that patients with depression have a significantly increased risk of CVD, and this association was more pronounced in women and individuals over 65 years of age (29). In a recent study, the degree of depression was also positively associated with the risk of CVD, with the more depressed patients being more likely to develop CVD (30). A recent meta-analysis showed that depression was a significant risk factor for CVD, which not only increases the incidence of stroke, myocardial infarction, and congestive heart failure, but also increases cardiovascular-related and all-cause mortality rates (31). These pieces of evidence suggest that depressed patients have a higher risk of nonfatal CVD and fatal CVD (32). Furthermore, being female, over 65 years of age, and having major depression may strengthen this association.

At present, the mechanisms associated with depression and CVD have not been fully elucidated, while some studies have suggested that inflammatory responses associated with the immune system are a comorbidity mechanism for depression and CVD (33). Some drugs with anti-inflammatory effects alleviate the clinical manifestations of patients with depression and CVD (34). There was also evidence that the cortisol-regulated stress inflammatory response in depressed patients is a crucial mechanism in the pathogenesis of CVD (35). Plasma cortisol levels are maintained high in depressed patients. The sensitivity of immune cells to cortisol is reduced due to the stimulation of persistently high cortisol levels, which leads to an increased susceptibility to CVD (35, 36). Moreover, cortisol has a vasoconstrictive effect and elevates blood pressure, and sustained high levels of cortisol are prone to vascular damage and plaque formation, resulting in an increased risk of CVD (37).

The topic of ANX and CVD has always been controversial. An early meta-analysis showed that ANX increased the risk of coronary heart disease and cardiovascular mortality in patients (38), but very few included studies in this analysis controlled for the depression variable. However, some researchers did not find a significant effect of ANX on CVD after adjusting for the interference of depression. In a Greek cohort study, Kyrou I et al. (39) found that depression increased cardiovascular events, whereas ANX had no significant effect on cardiovascular events. In a study of older Americans, Karlsen et al. (40) reported that ANX was not associated with coronary heart disease or cerebrovascular disease. Intriguingly, there is evidence that ANX may even be a protective factor for CVD when potential risk factors are excluded. In a cross-sectional study in Taiwan, Huang et al. (41) found that older adults with ANX but not depression had a significantly lower risk of developing coronary heart disease or hypertension than healthy individuals in the same age group. Langvik et al. (42) showed in a study that ANX reduced the risk of acute myocardial infarction when controlling for depression. Parker G et al. (43) noted that generalized anxiety disorder (GAD) significantly improved cardiac prognosis in patients with acute coronary syndromes. More and more researchers have realized that comorbid depression is an essential factor influencing the causal relationship between ANX and CVD. Batelaan et al. (44) conducted a meta-analysis of 14 clinical studies that excluded or controlled for cases of depression and found that ANX was strongly associated with increased cardiovascular risk. This meta-analysis supports our findings, and our results point to ANX as a potential risk factor for CVD.

The current research indicates that the mechanism of ANX-mediated CVD may be related to the stimulation of the nervous system and the induction of an inflammatory response. ANX induces CVD by stimulating the amygdala, hippocampus, medial prefrontal cortex, and the autonomic nervous system. First, ANX affects the survival and growth of hippocampal neurons, leading to hippocampal atrophy, which can cause a decrease in cardiac ejection fraction and increase N-terminal prohormone of brain natriuretic peptide (NT-proBNP) (45). Second, ANX stimulates the amygdala, which affects blood pressure and heart rate (46). Third, ANX affects cardiac autonomic regulation by stimulating the medial prefrontal cortex (47, 48). The decreased autonomic nervous system regulation of heart rate is one of the most critical risk factors for adverse cardiovascular events (49). It has also been shown that ANX may affect the normal functioning of the cardiovascular system through the sympathetic nervous system and the hypothalamic-pituitary-adrenal axis, thereby increasing the risk of myocardial ischemia, arrhythmia, and sudden cardiac death (50). Moreover, the inflammatory response is closely associated with MD and CVD and may play a role in the comorbidity of ANX and CVD (51). Related studies have shown that ANX patients have higher C-reactive protein (CRP), tumor necrosis factor-alpha (TNF-α), and interleukin-6 (IL-6) (52, 53). These inflammatory factors are also associated with developing diseases such as atherosclerosis, heart failure, and unstable angina pectoris (54).

At this stage, there are a few studies on the correlation between ASD and CVD, which may be related to the verbal communication deficits of ASD patients. Heffernan et al. (55) found that decreased physical activity and weight gain were prevalent in children with ASD, and these factors led to increased arterial stiffness, which adversely affected cardiovascular health. Related studies have shown that children with ASD have higher levels of homocysteine (HCY) (56), which are positively correlated with carotid artery intima-media thickness (CA-IMT), atherogenic index, left ventricular mass index (LVMI), and blood pressure (57) and are also strongly associated with the severity of heart failure (58). In addition, Imre Yetkin et al. (59) found that Childhood Autism Rating Scale (CARS) scores were positively correlated with vessel diameter, carotid intima-media thickness (cIMT), and intima-media thickness/diameter ratio (IDR) values in children with ASD, suggesting that CVD risk may increase with the severity of ASD. These pieces of evidence support ASD as a potential risk factor for CVD, which is consistent with our findings. Interestingly, a recent study has found that the mechanism of comorbidity between ASD and CVD may be related to dysregulated phosphate metabolism (60), which provides a new direction for future research.

We did not find a causal relationship of AN, ADHD, BD, OCD, and SCZ with CVD in this study, which is quite different from the results of previous cohort studies. The current mainstream view is that AN, ADHD, BD, OCD, and SCZ may be potential risk factors for CVD. A relevant study has shown that bradycardia and QT interval prolongation are the most common adverse cardiovascular events in patients with AN and that AN patients with concomitant eating disorders also have a higher risk of acute cardiac disease (61). A UK cohort study showed that ADHD significantly increased the risk of CVD, particularly hemorrhagic stroke, cardiac arrest, and atherosclerosis (62). A cross-sectional study found that patients with BD had a significantly higher risk of developing CVD over a 10-year period, which was more pronounced in patients with depressive phases (63). Isomura K et al. (64) noted that patients with OCD had a higher risk of CVD, which might be related to decreased parasympathetic function and abnormal sympathetic responses in OCD patients (65). Veeneman et al. (27) found that SCZ was associated with heart failure and cardiovascular death, which might be a potential risk factor for CVD. Although these pieces of evidence point to the possibility that MD such as AN, ADHD, BD, OCD, and SCZ may be associated with an increased risk of CVD, there have been some reports to the contrary. Kalla et al. (66) found that older patients with AN had a lower incidence of coronary heart disease and heart failure compared with the general population over 65. Kittel-Schneider et al. (67) concluded that there was currently insufficient evidence to argue for an association between ADHD and CVD, and potential cardiovascular risks from drug therapy could not be excluded (68). Therefore, the causal relationship of AN, ADHD, BD, OCD, and SCZ with CVD is inconclusive, and we expect more researchers to continue exploring it in the future.

In summary, this MR analysis supports the notion that depression, ANX, and ASD as potential risk factors for CVD. It underscores the connection between mental health and CVD, suggesting that proactive psychological screening and intervention may help to mitigate an individual's CVD risk. Therefore, we recommend that cardiovascular physicians endeavor to focus on psychological and mental well-being of their patients. Ultimately, these efforts have the potential to enhance the health and well-being of individuals, families, and communities by alleviating the burden of CVD and its related complications.

However, our study has some limitations. First, the data used in the study were exclusively derived from European populations, and the findings primarily explained the impact of MD on CVD risk in European populations. They did not apply to Asian, African, and Latino populations. Second, the study focused on the overall effect of MD on CVD and did not clarify the effect of MD on specific diseases such as coronary heart disease, hypertension, and heart failure. Third, this study only explained the effects of depression, ANX, and ASD on CVD but not reflect the specific effects of different levels of depression, ANX, and ASD on CVD. Fourth, although the sensitivity analysis suggested that the results were robust, the influence of study design, investigative population, and selection of specific genetic variants cannot be excluded. These factors may have contributed to the MR analysis not identifying an association between AN, ADHD, BD, OCD, SCZ, and CVD risk.

Given these limitations, we expect future studies to continue to improve: First, research centers should be established in Asia, Africa, and Latin America to explore the causal relationship between MD and CVD across different ethnicities. This will provide a more comprehensive data source for MR studies. Second, a more extensive stratified cohort study should be conducted to control for the variables of interest and explore the specific effects of different types and degrees of MD on the risk of CVD. Third, basic research is needed to explore the potential mechanisms by which depression, ANX, and ASD increase the risk of CVD in different populations.

## 5 Conclusion

This MR analysis showed that depression, ANX, and ASD were associated with an increased risk of CVD, whereas there was no causal relationship of AN, ADHD, BD, OCD, and SCZ with CVD. It implies that the active prevention and treatment of depression, ANX, and ASD may help reduce CVD risk. More studies are needed to explore the causal relationship and mechanism between MD and CVD.

## Data Availability

The original contributions presented in the study are included in the article/**Supplementary Material**, further inquiries can be directed to the corresponding author.
