# Supplementary material for: A Mendelian randomization study of the effect of mental disorders on cardiovascular disease
Source: Front Cardiovasc Med. 2024 Jun 3;11:1329463. doi: 10.3389/fcvm.2024.1329463 (PMC11180800; doi:10.3389/fcvm.2024.1329463)
Supplement: Supplementary file 1 [file Table1.docx]

**Supplementary Table S1.1** Characteristics of instrumental variables for ADHD

|  | **SNP** | **EA** | **OA** | **Samplesize** | **SE** | **β** | **id.exposure** | **EAF** | ***p* value** | **R^2^** | **F - statistic** |
| --- | --- | --- | --- | --- | --- | --- | --- | --- | --- | --- | --- |
| 1 | rs9661242 | A | G | 55,374 | 0.0135 | 0.0625991 | ieu-a-1183 | 0.5497 | 3.54E-06 | 0.001939965 | 107.6285285 |
| 2 | rs17531412 | A | G | 55,374 | 0.0145 | -0.105602 | ieu-a-1183 | 0.7087 | 3.27E-13 | 0.004604444 | 256.1366426 |
| 3 | rs1222063 | G | A | 55,374 | 0.0174 | 0.0962007 | ieu-a-1183 | 0.6292 | 3.22E-08 | 0.004318321 | 240.1511074 |
| 4 | rs756354 | C | G | 55,374 | 0.0138 | 0.0711953 | ieu-a-1183 | 0.3479 | 2.48E-07 | 0.002299859 | 127.641368 |
| 5 | rs76338508 | C | T | 55,374 | 0.0471 | -0.225095 | ieu-a-1183 | 0.9523 | 1.76E-06 | 0.004603137 | 256.063571 |
| 6 | rs9677504 | G | A | 55,374 | 0.0206 | 0.116903 | ieu-a-1183 | 0.0924 | 1.39E-08 | 0.002292175 | 127.2139092 |
| 7 | rs62259516 | C | T | 55,374 | 0.0286 | 0.143104 | ieu-a-1183 | 0.9443 | 5.63E-07 | 0.002154263 | 119.54338 |
| 8 | rs7634587 | A | G | 55,374 | 0.0137 | -0.0635005 | ieu-a-1183 | 0.6173 | 3.57E-06 | 0.001905193 | 105.6957291 |
| 9 | rs1513155 | G | A | 55,374 | 0.0182 | -0.0849045 | ieu-a-1183 | 0.8509 | 3.08E-06 | 0.001829143 | 101.4688888 |
| 10 | rs7631360 | A | G | 55,374 | 0.0137 | -0.0654974 | ieu-a-1183 | 0.5964 | 1.75E-06 | 0.002065223 | 114.5921747 |
| 11 | rs1272878 | T | C | 55,374 | 0.0177 | -0.0910009 | ieu-a-1183 | 0.2465 | 2.73E-07 | 0.003076249 | 170.8637047 |
| 12 | rs28411770 | T | C | 55,374 | 0.0151 | -0.0861043 | ieu-a-1183 | 0.3767 | 1.18E-08 | 0.003481548 | 193.4538108 |
| 13 | rs1484144 | T | C | 55,374 | 0.0133 | -0.0608975 | ieu-a-1183 | 0.5179 | 4.68E-06 | 0.001851876 | 102.7323403 |
| 14 | rs227378 | C | A | 55,374 | 0.0143 | 0.0740958 | ieu-a-1183 | 0.66 | 2.20E-07 | 0.002463996 | 136.7734059 |
| 15 | rs77216804 | A | T | 55,374 | 0.0216 | -0.103396 | ieu-a-1183 | 0.8797 | 1.69E-06 | 0.002262756 | 125.5774664 |
| 16 | rs433274 | T | C | 55,374 | 0.0199 | 0.0934978 | ieu-a-1183 | 0.8728 | 2.62E-06 | 0.001941041 | 107.6883298 |
| 17 | rs1077612 | C | T | 55,374 | 0.0165 | -0.0769026 | ieu-a-1183 | 0.2296 | 3.15E-06 | 0.002092186 | 116.0913834 |
| 18 | rs6933023 | C | T | 55,374 | 0.0133 | 0.066602 | ieu-a-1183 | 0.5437 | 5.51E-07 | 0.002200971 | 122.1410003 |
| 19 | rs4839923 | G | A | 55,374 | 0.0133 | 0.0666956 | ieu-a-1183 | 0.4085 | 5.31E-07 | 0.002149667 | 119.2877857 |
| 20 | rs180822580 | G | A | 55,374 | 0.0324 | -0.155298 | ieu-a-1183 | 0.0586 | 1.64E-06 | 0.00266093 | 147.7341538 |
| 21 | rs10262192 | G | A | 55,374 | 0.0132 | 0.073204 | ieu-a-1183 | 0.4264 | 2.93E-08 | 0.002621356 | 145.531198 |
| 22 | rs28452470 | T | A | 55,374 | 0.0138 | 0.074003 | ieu-a-1183 | 0.3678 | 8.21E-08 | 0.0025468 | 141.3814874 |
| 23 | rs7459616 | G | C | 55,374 | 0.0135 | 0.064701 | ieu-a-1183 | 0.4503 | 1.65E-06 | 0.002072429 | 114.9928544 |
| 24 | rs74760947 | A | G | 55,374 | 0.0317 | 0.179797 | ieu-a-1183 | 0.9404 | 1.41E-08 | 0.003623713 | 201.38197 |
| 25 | rs4735168 | G | C | 55,374 | 0.0148 | -0.0732053 | ieu-a-1183 | 0.3151 | 7.56E-07 | 0.00231308 | 128.3768 |
| 26 | rs7824062 | G | A | 55,374 | 0.0134 | 0.0619978 | ieu-a-1183 | 0.4682 | 3.72E-06 | 0.00191409 | 106.1902348 |
| 27 | rs10965173 | A | G | 55,374 | 0.0167 | -0.0783027 | ieu-a-1183 | 0.7823 | 2.75E-06 | 0.002088407 | 115.8813037 |
| 28 | rs143912172 | A | C | 55,374 | 0.029 | -0.138796 | ieu-a-1183 | 0.9354 | 1.70E-06 | 0.002328165 | 129.2159956 |
| 29 | rs9665567 | A | T | 55,374 | 0.0136 | -0.0696007 | ieu-a-1183 | 0.4115 | 3.09E-07 | 0.002346246 | 130.2218577 |
| 30 | rs10400180 | T | G | 55,374 | 0.0163 | -0.0925974 | ieu-a-1183 | 0.2306 | 1.34E-08 | 0.003042559 | 168.9867513 |
| 31 | rs28633403 | G | A | 55,374 | 0.0142 | 0.0712038 | ieu-a-1183 | 0.5258 | 5.32E-07 | 0.002528241 | 140.3485959 |
| 32 | rs11245604 | G | A | 55,374 | 0.0253 | 0.129299 | ieu-a-1183 | 0.0984 | 3.21E-07 | 0.002966397 | 164.7440522 |
| 33 | rs61896068 | G | A | 55,374 | 0.0161 | 0.0744022 | ieu-a-1183 | 0.2475 | 3.81E-06 | 0.002061974 | 114.4115573 |
| 34 | rs7989860 | G | A | 55,374 | 0.0132 | 0.0639978 | ieu-a-1183 | 0.5567 | 1.25E-06 | 0.002021525 | 112.1626015 |
| 35 | rs2243517 | T | C | 55,374 | 0.0136 | 0.072001 | ieu-a-1183 | 0.34 | 1.20E-07 | 0.002326644 | 129.1313647 |
| 36 | rs10444728 | G | A | 55,374 | 0.0139 | -0.0679042 | ieu-a-1183 | 0.5726 | 1.03E-06 | 0.002256883 | 125.2508267 |
| 37 | rs60798171 | T | G | 55,374 | 0.0155 | 0.0733021 | ieu-a-1183 | 0.2366 | 2.25E-06 | 0.001941019 | 107.6871115 |
| 38 | rs1859057 | C | G | 55,374 | 0.0135 | -0.0639039 | ieu-a-1183 | 0.5358 | 2.21E-06 | 0.002031387 | 112.7108943 |
| 39 | rs8058677 | C | T | 55,374 | 0.0135 | 0.0680978 | ieu-a-1183 | 0.6243 | 4.55E-07 | 0.002175358 | 120.7165108 |
| 40 | rs212178 | G | A | 55,374 | 0.02 | -0.1154 | ieu-a-1183 | 0.1014 | 7.93E-09 | 0.002426867 | 134.7073984 |
| 41 | rs4144756 | G | A | 55,374 | 0.0145 | 0.0763961 | ieu-a-1183 | 0.4235 | 1.37E-07 | 0.00284987 | 158.254023 |
| 42 | rs2144782 | C | T | 55,374 | 0.0143 | 0.074003 | ieu-a-1183 | 0.3728 | 2.28E-07 | 0.002561006 | 142.1721295 |
| 43 | rs11698378 | T | C | 55,374 | 0.0175 | 0.0827948 | ieu-a-1183 | 0.2078 | 2.23E-06 | 0.002256922 | 125.2529567 |
| 44 | rs6063848 | G | T | 55,374 | 0.0148 | 0.0705983 | ieu-a-1183 | 0.3539 | 1.84E-06 | 0.002279286 | 126.4969364 |
| 45 | rs992936 | T | C | 55,374 | 0.0135 | -0.0731018 | ieu-a-1183 | 0.5457 | 6.13E-08 | 0.002649615 | 147.1042698 |
| 46 | rs2835344 | C | T | 55,374 | 0.0158 | -0.0754997 | ieu-a-1183 | 0.7406 | 1.77E-06 | 0.002190151 | 121.5392497 |

SNP, single nucleotide polymorphism; EA, effect allele; OA, other allele; EAF, effect allele frequency; SE, standard error; ADHD, attention deficit hyperactivity disorder.

**Supplementary Table S1.2** Characteristics of instrumental variables for AN

|  | **SNP** | **EA** | **OA** | **Samplesize** | **SE** | **β** | **id.exposure** | **EAF** | ***p* value** | **R^2^** | **F - statistic** |
| --- | --- | --- | --- | --- | --- | --- | --- | --- | --- | --- | --- |
| 1 | rs145106667 | G | A | 14,477 | 0.1483 | 0.686291 | ieu-a-1186 | 0.9891 | 3.70E-06 | 0.01015578 | 148.513189 |
| 2 | rs75307524 | G | A | 14,477 | 0.101 | 0.474397 | ieu-a-1186 | 0.9751 | 2.64E-06 | 0.010928546 | 159.9385932 |
| 3 | rs61831281 | C | G | 14,477 | 0.0892 | 0.416801 | ieu-a-1186 | 0.0328 | 2.97E-06 | 0.011022437 | 161.3280058 |
| 4 | rs111925923 | T | C | 14,477 | 0.089 | 0.409497 | ieu-a-1186 | 0.9622 | 4.20E-06 | 0.012197999 | 178.7463852 |
| 5 | rs13125782 | C | T | 14,477 | 0.0356 | -0.174802 | ieu-a-1186 | 0.7873 | 9.10E-07 | 0.010233649 | 149.6636798 |
| 6 | rs200312312 | C | T | 14,477 | 0.0335 | -0.180603 | ieu-a-1186 | 0.2992 | 7.00E-08 | 0.013678409 | 200.7407908 |
| 7 | rs79350580 | T | C | 14,477 | 0.0666 | 0.309196 | ieu-a-1186 | 0.9553 | 3.44E-06 | 0.00816479 | 119.1582404 |
| 8 | rs75544652 | T | C | 14,477 | 0.112 | 0.5223 | ieu-a-1186 | 0.9881 | 3.11E-06 | 0.006415314 | 93.46125129 |
| 9 | rs1894792 | T | C | 14,477 | 0.0364 | 0.172599 | ieu-a-1186 | 0.7306 | 2.12E-06 | 0.011726916 | 171.7613363 |
| 10 | rs4622308 | T | C | 14,477 | 0.0307 | -0.180096 | ieu-a-1186 | 0.5527 | 4.46E-09 | 0.016037124 | 235.920865 |
| 11 | rs4763166 | G | A | 14,477 | 0.0298 | -0.152403 | ieu-a-1186 | 0.5885 | 3.15E-07 | 0.011249503 | 164.6892273 |
| 12 | rs117957029 | C | T | 14,477 | 0.1024 | 0.536502 | ieu-a-1186 | 0.0189 | 1.61E-07 | 0.010674506 | 156.1806183 |
| 13 | rs2345557 | T | G | 14,477 | 0.051 | 0.237299 | ieu-a-1186 | 0.9254 | 3.27E-06 | 0.007774816 | 113.422303 |
| 14 | rs75115477 | G | A | 14,477 | 0.0593 | 0.277204 | ieu-a-1186 | 0.9334 | 2.95E-06 | 0.009553687 | 139.6235383 |

SNP, single nucleotide polymorphism; EA, effect allele; OA, other allele; EAF, effect allele frequency; SE, standard error; AN, anorexia nervosa.

**Supplementary Table S1.3** Characteristics of instrumental variables for ANX

|  | **SNP** | **EA** | **OA** | **Samplesize** | **SE** | **β** | **id.exposure** | **EAF** | ***p* value** | **R^2^** | **F - statistic** |
| --- | --- | --- | --- | --- | --- | --- | --- | --- | --- | --- | --- |
| 1 | rs7555230 | C | T | 362,239 | 0.0101682 | 0.0466512 | finngen_R9_F5_ALLANXIOUS | 0.339912 | 4.48E-06 | 0.000976616 | 354.1123911 |
| 2 | rs56258708 | G | A | 362,239 | 0.0119959 | 0.0558644 | finngen_R9_F5_ALLANXIOUS | 0.200018 | 3.21E-06 | 0.000998733 | 362.1398674 |
| 3 | rs61820769 | T | C | 362,239 | 0.026501 | -0.13383 | finngen_R9_F5_ALLANXIOUS | 0.0373594 | 4.42E-07 | 0.001288253 | 467.2546895 |
| 4 | rs4330896 | A | G | 362,239 | 0.0107951 | 0.0563465 | finngen_R9_F5_ALLANXIOUS | 0.71513 | 1.79E-07 | 0.001293587 | 469.1919667 |
| 5 | rs79634932 | G | C | 362,239 | 0.0138332 | 0.0678968 | finngen_R9_F5_ALLANXIOUS | 0.141127 | 9.19E-07 | 0.001117552 | 405.2715287 |
| 6 | rs2215964 | C | T | 362,239 | 0.0110657 | -0.0534287 | finngen_R9_F5_ALLANXIOUS | 0.26387 | 1.38E-06 | 0.00110898 | 402.1596035 |
| 7 | rs6751342 | C | A | 362,239 | 0.0101762 | -0.0481313 | finngen_R9_F5_ALLANXIOUS | 0.350451 | 2.25E-06 | 0.001054689 | 382.4507327 |
| 8 | rs55945133 | T | C | 362,239 | 0.0176946 | 0.0878536 | finngen_R9_F5_ALLANXIOUS | 0.0792069 | 6.87E-07 | 0.001125834 | 408.2782269 |
| 9 | rs7570682 | A | G | 362,239 | 0.0112745 | 0.0555894 | finngen_R9_F5_ALLANXIOUS | 0.237403 | 8.20E-07 | 0.00111891 | 405.7647174 |
| 10 | rs12614303 | T | C | 362,239 | 0.00972451 | -0.0476777 | finngen_R9_F5_ALLANXIOUS | 0.46619 | 9.45E-07 | 0.001131385 | 410.2935487 |
| 11 | rs72967615 | T | G | 362,239 | 0.0247645 | 0.114426 | finngen_R9_F5_ALLANXIOUS | 0.0382078 | 3.83E-06 | 0.000962305 | 348.918217 |
| 12 | rs869208 | A | G | 362,239 | 0.0120081 | -0.056345 | finngen_R9_F5_ALLANXIOUS | 0.213181 | 2.70E-06 | 0.001065036 | 386.2065987 |
| 13 | rs189295234 | A | G | 362,239 | 0.0475714 | 0.219074 | finngen_R9_F5_ALLANXIOUS | 0.0099448 | 4.12E-06 | 0.000945077 | 342.6656554 |
| 14 | rs145525394 | C | T | 362,239 | 0.0185721 | -0.0963933 | finngen_R9_F5_ALLANXIOUS | 0.078006 | 2.10E-07 | 0.001336533 | 484.7897645 |
| 15 | rs12498967 | A | G | 362,239 | 0.010545 | 0.052111 | finngen_R9_F5_ALLANXIOUS | 0.29583 | 7.74E-07 | 0.00113138 | 410.2919359 |
| 16 | rs72723168 | T | C | 362,239 | 0.0255836 | 0.117612 | finngen_R9_F5_ALLANXIOUS | 0.0363391 | 4.28E-06 | 0.000968795 | 351.2735306 |
| 17 | rs147631399 | G | A | 362,239 | 0.0586524 | 0.28299 | finngen_R9_F5_ALLANXIOUS | 0.00650815 | 1.40E-06 | 0.001035605 | 375.5232541 |
| 18 | rs250839 | G | T | 362,239 | 0.0106807 | 0.05162 | finngen_R9_F5_ALLANXIOUS | 0.697077 | 1.34E-06 | 0.001125328 | 408.0945604 |
| 19 | rs6450476 | G | A | 362,239 | 0.0107966 | 0.0564773 | finngen_R9_F5_ALLANXIOUS | 0.712821 | 1.69E-07 | 0.001305903 | 473.6650474 |
| 20 | rs584748 | T | C | 362,239 | 0.0099767 | -0.0469784 | finngen_R9_F5_ALLANXIOUS | 0.624105 | 2.49E-06 | 0.001035501 | 375.4857011 |
| 21 | rs7379967 | A | G | 362,239 | 0.00973847 | 0.0454037 | finngen_R9_F5_ALLANXIOUS | 0.482247 | 3.13E-06 | 0.001029449 | 373.288635 |
| 22 | rs10078559 | G | T | 362,239 | 0.0294875 | 0.149171 | finngen_R9_F5_ALLANXIOUS | 0.027656 | 4.22E-07 | 0.001196763 | 434.0312207 |
| 23 | rs2397085 | C | T | 362,239 | 0.018698 | -0.0874888 | finngen_R9_F5_ALLANXIOUS | 0.0743888 | 2.88E-06 | 0.001054074 | 382.2274715 |
| 24 | rs1059307 | T | G | 362,239 | 0.00968404 | 0.0463918 | finngen_R9_F5_ALLANXIOUS | 0.509267 | 1.66E-06 | 0.00107573 | 390.0888033 |
| 25 | rs78175056 | C | T | 362,239 | 0.0100688 | 0.0506886 | finngen_R9_F5_ALLANXIOUS | 0.356408 | 4.80E-07 | 0.001178715 | 427.4779212 |
| 26 | rs13244325 | G | T | 362,239 | 0.013909 | -0.0636689 | finngen_R9_F5_ALLANXIOUS | 0.144742 | 4.70E-06 | 0.001003636 | 363.9194811 |
| 27 | rs6965423 | C | T | 362,239 | 0.00968997 | 0.045814 | finngen_R9_F5_ALLANXIOUS | 0.500058 | 2.27E-06 | 0.001049461 | 380.5530828 |
| 28 | rs11243619 | T | G | 362,239 | 0.0123952 | -0.0592033 | finngen_R9_F5_ALLANXIOUS | 0.195791 | 1.79E-06 | 0.001103782 | 400.2726524 |
| 29 | rs4494021 | C | T | 362,239 | 0.00997619 | 0.0466473 | finngen_R9_F5_ALLANXIOUS | 0.386257 | 2.93E-06 | 0.001031682 | 374.0994131 |
| 30 | rs72805914 | A | C | 362,239 | 0.0304827 | 0.152923 | finngen_R9_F5_ALLANXIOUS | 0.0241586 | 5.26E-07 | 0.001102622 | 399.8513362 |
| 31 | rs2051523 | A | G | 362,239 | 0.0132834 | 0.0688427 | finngen_R9_F5_ALLANXIOUS | 0.153346 | 2.19E-07 | 0.001230621 | 446.3255937 |
| 32 | rs117632798 | T | A | 362,239 | 0.0193497 | -0.101036 | finngen_R9_F5_ALLANXIOUS | 0.0711162 | 1.77E-07 | 0.00134869 | 489.2052938 |
| 33 | rs1119988 | C | T | 362,239 | 0.00986764 | -0.0487858 | finngen_R9_F5_ALLANXIOUS | 0.415349 | 7.65E-07 | 0.001155917 | 419.2005377 |
| 34 | rs4772087 | T | C | 362,239 | 0.00983936 | 0.0575959 | finngen_R9_F5_ALLANXIOUS | 0.415579 | 4.81E-09 | 0.00161136 | 584.6362203 |
| 35 | rs2281530 | C | T | 362,239 | 0.00975356 | -0.0447306 | finngen_R9_F5_ALLANXIOUS | 0.466918 | 4.52E-06 | 0.000996034 | 361.1600247 |
| 36 | rs2756119 | A | G | 362,239 | 0.0100408 | -0.0507645 | finngen_R9_F5_ALLANXIOUS | 0.382851 | 4.29E-07 | 0.001217783 | 441.6640437 |
| 37 | rs11857461 | T | C | 362,239 | 0.00968185 | 0.0469825 | finngen_R9_F5_ALLANXIOUS | 0.497338 | 1.22E-06 | 0.001103646 | 400.2232549 |
| 38 | rs1036810 | C | G | 362,239 | 0.00975224 | -0.0494732 | finngen_R9_F5_ALLANXIOUS | 0.545679 | 3.92E-07 | 0.001213585 | 440.1393864 |
| 39 | rs34832985 | G | A | 362,239 | 0.0099834 | -0.054433 | finngen_R9_F5_ALLANXIOUS | 0.620932 | 4.97E-08 | 0.001394812 | 505.9582631 |
| 40 | rs215894 | A | T | 362,239 | 0.0100136 | 0.0510017 | finngen_R9_F5_ALLANXIOUS | 0.371343 | 3.52E-07 | 0.001214474 | 440.4624256 |
| 41 | rs554005300 | A | T | 362,239 | 0.0319341 | 0.155329 | finngen_R9_F5_ALLANXIOUS | 0.0232205 | 1.15E-06 | 0.001094468 | 396.8913025 |
| 42 | rs57852066 | A | G | 362,239 | 0.0217339 | 0.103968 | finngen_R9_F5_ALLANXIOUS | 0.0492355 | 1.72E-06 | 0.001012 | 366.9553487 |
| 43 | rs28478424 | G | T | 362,239 | 0.0151606 | 0.0784687 | finngen_R9_F5_ALLANXIOUS | 0.112089 | 2.27E-07 | 0.001225619 | 444.5092023 |
| 44 | rs62126622 | G | T | 362,239 | 0.0469004 | 0.220803 | finngen_R9_F5_ALLANXIOUS | 0.0101068 | 2.50E-06 | 0.000975533 | 353.7191979 |

SNP, single nucleotide polymorphism; EA, effect allele; OA, other allele; EAF, effect allele frequency; SE, standard error; ANX, anxiety disorder.

**Supplementary Table S1.4** Characteristics of instrumental variables for ASD

|  | **SNP** | **EA** | **OA** | **Samplesize** | **SE** | **β** | **id.exposure** | **EAF** | ***p* value** | **R^2^** | **F - statistic** |
| --- | --- | --- | --- | --- | --- | --- | --- | --- | --- | --- | --- |
| 1 | rs2391769 | G | A | 46,351 | 0.0145 | 0.0769026 | ieu-a-1185 | 0.33 | 1.14E-07 | 0.002615175 | 121.5285725 |
| 2 | rs6701243 | C | A | 46,351 | 0.0144 | -0.0735014 | ieu-a-1185 | 0.6412 | 3.32E-07 | 0.002485806 | 115.5017197 |
| 3 | rs11185408 | A | G | 46,351 | 0.0138 | -0.0686965 | ieu-a-1185 | 0.5089 | 6.42E-07 | 0.002358857 | 109.5891654 |
| 4 | rs78653484 | T | C | 46,351 | 0.0385 | -0.176296 | ieu-a-1185 | 0.9493 | 4.67E-06 | 0.002991757 | 139.0810539 |
| 5 | rs6692705 | G | A | 46,351 | 0.0141 | -0.0656005 | ieu-a-1185 | 0.3907 | 3.28E-06 | 0.002048891 | 95.15902586 |
| 6 | rs79940520 | G | A | 46,351 | 0.0207 | 0.0953992 | ieu-a-1185 | 0.8519 | 4.05E-06 | 0.002296482 | 106.6846663 |
| 7 | rs2635182 | T | C | 46,351 | 0.014 | 0.0669014 | ieu-a-1185 | 0.5278 | 1.76E-06 | 0.002230981 | 103.6349232 |
| 8 | rs9366877 | G | A | 46,351 | 0.0139 | -0.0684994 | ieu-a-1185 | 0.5726 | 8.31E-07 | 0.002296621 | 106.6911307 |
| 9 | rs16879023 | A | G | 46,351 | 0.0201 | -0.0957953 | ieu-a-1185 | 0.1521 | 1.88E-06 | 0.002366967 | 109.9668529 |
| 10 | rs12203328 | C | G | 46,351 | 0.0153 | 0.0697033 | ieu-a-1185 | 0.2445 | 5.22E-06 | 0.00179494 | 83.34328391 |
| 11 | rs740883 | T | A | 46,351 | 0.0238 | 0.113695 | ieu-a-1185 | 0.0974 | 1.78E-06 | 0.00227283 | 105.5833855 |
| 12 | rs72934503 | G | A | 46,351 | 0.0141 | 0.0704976 | ieu-a-1185 | 0.5169 | 5.74E-07 | 0.002482117 | 115.329898 |
| 13 | rs9389208 | T | C | 46,351 | 0.0144 | 0.0672006 | ieu-a-1185 | 0.6233 | 3.06E-06 | 0.00212065 | 98.49889926 |
| 14 | rs7783557 | C | T | 46,351 | 0.0146 | -0.0670042 | ieu-a-1185 | 0.3211 | 4.45E-06 | 0.001957403 | 90.90158444 |
| 15 | rs111931861 | G | A | 46,351 | 0.0409 | 0.216901 | ieu-a-1185 | 0.9433 | 1.14E-07 | 0.005032526 | 234.4323183 |
| 16 | rs76397219 | G | A | 46,351 | 0.0303 | 0.140297 | ieu-a-1185 | 0.9175 | 3.65E-06 | 0.002979798 | 138.5234171 |
| 17 | rs10110094 | G | A | 46,351 | 0.0191 | -0.0906996 | ieu-a-1185 | 0.1451 | 2.05E-06 | 0.002040908 | 94.78750634 |
| 18 | rs28729902 | G | A | 46,351 | 0.0178 | 0.0839035 | ieu-a-1185 | 0.8042 | 2.43E-06 | 0.002217006 | 102.9843372 |
| 19 | rs45595836 | T | C | 46,351 | 0.0272 | 0.138996 | ieu-a-1185 | 0.9066 | 3.22E-07 | 0.003271879 | 152.1461084 |
| 20 | rs141319505 | G | A | 46,351 | 0.061 | -0.290698 | ieu-a-1185 | 0.9692 | 1.88E-06 | 0.005045198 | 235.0256277 |
| 21 | rs78827416 | A | G | 46,351 | 0.0266 | 0.130502 | ieu-a-1185 | 0.0746 | 9.29E-07 | 0.002351433 | 109.243458 |
| 22 | rs4750990 | C | T | 46,351 | 0.0141 | 0.0680968 | ieu-a-1185 | 0.3857 | 1.37E-06 | 0.002197422 | 102.07263 |
| 23 | rs644552 | A | G | 46,351 | 0.0346 | 0.159403 | ieu-a-1185 | 0.0626 | 4.08E-06 | 0.0029821 | 138.6307818 |
| 24 | rs35404050 | T | C | 46,351 | 0.0176 | 0.0843044 | ieu-a-1185 | 0.7883 | 1.67E-06 | 0.002372154 | 110.208393 |
| 25 | rs77691144 | C | T | 46,351 | 0.0435 | 0.207406 | ieu-a-1185 | 0.0318 | 1.86E-06 | 0.002648896 | 123.0997359 |
| 26 | rs141455452 | G | T | 46,351 | 0.0159 | -0.0784044 | ieu-a-1185 | 0.4334 | 8.18E-07 | 0.003019092 | 140.3556407 |
| 27 | rs292441 | A | G | 46,351 | 0.0149 | -0.0724954 | ieu-a-1185 | 0.327 | 1.14E-06 | 0.002313203 | 107.4632218 |
| 28 | rs149923766 | G | T | 46,351 | 0.0484 | 0.237306 | ieu-a-1185 | 0.0119 | 9.44E-07 | 0.001324327 | 61.46263737 |
| 29 | rs2224274 | T | C | 46,351 | 0.0138 | 0.0709989 | ieu-a-1185 | 0.498 | 2.68E-07 | 0.002520382 | 117.1123333 |
| 30 | rs910805 | A | G | 46,351 | 0.016 | -0.0956963 | ieu-a-1185 | 0.7644 | 2.22E-09 | 0.003298498 | 153.3880439 |
| 31 | rs144911765 | C | T | 46,351 | 0.0403 | 0.190096 | ieu-a-1185 | 0.0268 | 2.39E-06 | 0.001885006 | 87.53316583 |

SNP, single nucleotide polymorphism; EA, effect allele; OA, other allele; EAF, effect allele frequency; SE, standard error; ASD, autism spectrum disorder.

**Supplementary Table S1.5** Characteristics of instrumental variables for BD

|  | **SNP** | **EA** | **OA** | **Samplesize** | **SE** | **β** | **id.exposure** | **EAF** | ***p* value** | **R^2^** | **F - statistic** |
| --- | --- | --- | --- | --- | --- | --- | --- | --- | --- | --- | --- |
| 1 | rs147538909 | T | C | 51,710 | 0.0623 | 0.310502 | ieu-b-41 | 0.0367137 | 6.23E-07 | 0.00681934 | 355.0355357 |
| 2 | rs814197 | G | T | 51,710 | 0.0134 | 0.070498 | ieu-b-41 | 0.455084 | 1.43E-07 | 0.002464931 | 127.7715851 |
| 3 | rs12563424 | C | T | 51,710 | 0.0139 | 0.069704 | ieu-b-41 | 0.360329 | 5.31E-07 | 0.002239759 | 116.0734311 |
| 4 | rs12135727 | T | C | 51,710 | 0.0165 | -0.076104 | ieu-b-41 | 0.215064 | 3.98E-06 | 0.001955451 | 101.3105663 |
| 5 | rs12063329 | C | T | 51,710 | 0.0181 | -0.091101 | ieu-b-41 | 0.16449 | 4.82E-07 | 0.002281221 | 118.2270983 |
| 6 | rs80148877 | C | T | 51,710 | 0.0309 | 0.150497 | ieu-b-41 | 0.0817551 | 1.11E-06 | 0.003400627 | 176.4396514 |
| 7 | rs2877947 | C | T | 51,710 | 0.0191 | 0.091797 | ieu-b-41 | 0.143329 | 1.54E-06 | 0.002069356 | 107.2241197 |
| 8 | rs111725205 | G | C | 51,710 | 0.0179 | 0.089301 | ieu-b-41 | 0.170117 | 6.07E-07 | 0.002251683 | 116.6927729 |
| 9 | rs2339519 | A | G | 51,710 | 0.0136 | 0.062599 | ieu-b-41 | 0.454117 | 4.17E-06 | 0.001942818 | 100.6547866 |
| 10 | rs57681866 | A | G | 51,710 | 0.0296 | -0.161402 | ieu-b-41 | 0.0579578 | 4.96E-08 | 0.002844658 | 147.5111978 |
| 11 | rs3911862 | G | A | 51,710 | 0.0134 | -0.0635 | ieu-b-41 | 0.547309 | 2.15E-06 | 0.001998076 | 103.5233358 |
| 12 | rs2314398 | G | C | 51,710 | 0.0144 | -0.084102 | ieu-b-41 | 0.319554 | 5.21E-09 | 0.003075959 | 159.5424429 |
| 13 | rs12621381 | C | A | 51,710 | 0.0139 | 0.067797 | ieu-b-41 | 0.403117 | 1.07E-06 | 0.002211929 | 114.6279975 |
| 14 | rs72927105 | A | T | 51,710 | 0.0246 | 0.116004 | ieu-b-41 | 0.0883667 | 2.41E-06 | 0.002168127 | 112.3531131 |
| 15 | rs17183814 | A | G | 51,710 | 0.0268 | -0.140896 | ieu-b-41 | 0.0721056 | 1.46E-07 | 0.002656409 | 137.7234238 |
| 16 | rs12474837 | C | A | 51,710 | 0.0287 | 0.138802 | ieu-b-41 | 0.0577551 | 1.32E-06 | 0.00209689 | 108.6538058 |
| 17 | rs13003404 | C | T | 51,710 | 0.0137 | 0.0727 | ieu-b-41 | 0.593478 | 1.12E-07 | 0.002550278 | 132.2069304 |
| 18 | rs16842765 | A | G | 51,710 | 0.0145 | -0.066599 | ieu-b-41 | 0.313883 | 4.37E-06 | 0.001910431 | 98.97365537 |
| 19 | rs735931 | G | A | 51,710 | 0.0138 | -0.066901 | ieu-b-41 | 0.505703 | 1.25E-06 | 0.002237581 | 115.9602965 |
| 20 | rs12639551 | C | G | 51,710 | 0.0225 | 0.1028 | ieu-b-41 | 0.121936 | 4.90E-06 | 0.002262947 | 117.2778458 |
| 21 | rs9834970 | C | T | 51,710 | 0.0134 | 0.101003 | ieu-b-41 | 0.501233 | 4.79E-14 | 0.005100772 | 265.1029475 |
| 22 | rs6767302 | G | A | 51,710 | 0.0135 | -0.0703 | ieu-b-41 | 0.501522 | 1.91E-07 | 0.002471022 | 128.0881195 |
| 23 | rs7613933 | A | G | 51,710 | 0.0137 | -0.066599 | ieu-b-41 | 0.485309 | 1.17E-06 | 0.002215799 | 114.8289645 |
| 24 | rs6782817 | A | C | 51,710 | 0.0183 | -0.084905 | ieu-b-41 | 0.165671 | 3.49E-06 | 0.001992876 | 103.2534235 |
| 25 | rs34568676 | A | G | 51,710 | 0.0159 | 0.074402 | ieu-b-41 | 0.243329 | 2.88E-06 | 0.002038451 | 105.6195002 |
| 26 | rs11097326 | A | G | 51,710 | 0.0149 | 0.071399 | ieu-b-41 | 0.298297 | 1.65E-06 | 0.002134108 | 110.5864801 |
| 27 | rs2635253 | T | C | 51,710 | 0.0135 | 0.067902 | ieu-b-41 | 0.497297 | 4.91E-07 | 0.002305273 | 119.4765045 |
| 28 | rs6829845 | G | A | 51,710 | 0.0142 | -0.072897 | ieu-b-41 | 0.668064 | 2.84E-07 | 0.002356795 | 122.1530243 |
| 29 | rs11724116 | T | C | 51,710 | 0.0188 | -0.104095 | ieu-b-41 | 0.155277 | 3.08E-08 | 0.00284257 | 147.4026177 |
| 30 | rs28565152 | A | G | 51,710 | 0.0158 | 0.080298 | ieu-b-41 | 0.251329 | 3.73E-07 | 0.00242646 | 125.7725555 |
| 31 | rs185308 | T | C | 51,710 | 0.0138 | 0.068499 | ieu-b-41 | 0.391691 | 6.92E-07 | 0.002235972 | 115.8767192 |
| 32 | rs10075788 | G | A | 51,710 | 0.0143 | 0.071195 | ieu-b-41 | 0.361904 | 6.40E-07 | 0.002341038 | 121.3344209 |
| 33 | rs329319 | G | A | 51,710 | 0.0139 | -0.078802 | ieu-b-41 | 0.565703 | 1.43E-08 | 0.003051264 | 158.2576455 |
| 34 | rs17566118 | T | C | 51,710 | 0.0152 | -0.072399 | ieu-b-41 | 0.267096 | 1.91E-06 | 0.002052152 | 106.3309028 |
| 35 | rs7708829 | G | A | 51,710 | 0.0135 | -0.064701 | ieu-b-41 | 0.452096 | 1.65E-06 | 0.002073897 | 107.4599105 |
| 36 | rs36034627 | T | G | 51,710 | 0.0171 | -0.078697 | ieu-b-41 | 0.188671 | 4.18E-06 | 0.001896044 | 98.22690584 |
| 37 | rs55648125 | G | A | 51,710 | 0.0215 | 0.117096 | ieu-b-41 | 0.108936 | 5.14E-08 | 0.002661917 | 138.0097519 |
| 38 | rs62433108 | G | T | 51,710 | 0.0148 | 0.0683 | ieu-b-41 | 0.28651 | 3.93E-06 | 0.001907212 | 98.8065877 |
| 39 | rs9371601 | T | G | 51,710 | 0.0139 | 0.066499 | ieu-b-41 | 0.354904 | 1.72E-06 | 0.002024862 | 104.9140098 |
| 40 | rs10455979 | G | C | 51,710 | 0.0137 | 0.075004 | ieu-b-41 | 0.473478 | 4.38E-08 | 0.002804886 | 145.4429822 |
| 41 | rs10950456 | A | G | 51,710 | 0.0138 | 0.065104 | ieu-b-41 | 0.576723 | 2.39E-06 | 0.002069366 | 107.2246576 |
| 42 | rs12538191 | A | G | 51,710 | 0.0182 | -0.095795 | ieu-b-41 | 0.239851 | 1.41E-07 | 0.003346231 | 173.6078537 |
| 43 | rs13231398 | C | G | 51,710 | 0.0219 | -0.1207 | ieu-b-41 | 0.107064 | 3.56E-08 | 0.002785533 | 144.436682 |
| 44 | rs77516904 | G | A | 51,710 | 0.0355 | 0.170006 | ieu-b-41 | 0.0385743 | 1.68E-06 | 0.002143741 | 111.0866907 |
| 45 | rs12703284 | G | C | 51,710 | 0.0163 | 0.086604 | ieu-b-41 | 0.782117 | 1.08E-07 | 0.002556236 | 132.516602 |
| 46 | rs143577122 | T | C | 51,710 | 0.0395 | -0.184199 | ieu-b-41 | 0.0444289 | 3.11E-06 | 0.002880933 | 149.397668 |
| 47 | rs12677998 | A | G | 51,710 | 0.0247 | -0.120102 | ieu-b-41 | 0.1053 | 1.16E-06 | 0.002717918 | 140.9210943 |
| 48 | rs10092482 | T | C | 51,710 | 0.0172 | 0.083799 | ieu-b-41 | 0.18351 | 1.10E-06 | 0.002104351 | 109.0412658 |
| 49 | rs10106152 | T | C | 51,710 | 0.0186 | -0.090001 | ieu-b-41 | 0.300883 | 1.31E-06 | 0.003407785 | 176.8122817 |
| 50 | rs61088439 | A | T | 51,710 | 0.0183 | 0.0966 | ieu-b-41 | 0.157936 | 1.30E-07 | 0.00248205 | 128.6611724 |
| 51 | rs2597374 | T | A | 51,710 | 0.0147 | -0.0692 | ieu-b-41 | 0.30949 | 2.51E-06 | 0.002046722 | 106.0489344 |
| 52 | rs189574365 | A | C | 51,710 | 0.0435 | 0.206803 | ieu-b-41 | 0.0261498 | 1.99E-06 | 0.002178232 | 112.8779102 |
| 53 | rs10120508 | A | G | 51,710 | 0.0148 | -0.0727 | ieu-b-41 | 0.711703 | 9.01E-07 | 0.002168891 | 112.3927862 |
| 54 | rs7915021 | T | C | 51,710 | 0.02 | -0.099202 | ieu-b-41 | 0.131671 | 7.05E-07 | 0.002250325 | 116.6222555 |
| 55 | rs10994318 | C | G | 51,710 | 0.0279 | 0.1409 | ieu-b-41 | 0.0601944 | 4.41E-07 | 0.002246188 | 116.4073613 |
| 56 | rs7916271 | T | C | 51,710 | 0.0141 | -0.070401 | ieu-b-41 | 0.490703 | 5.95E-07 | 0.002477294 | 128.4140173 |
| 57 | rs4595478 | C | T | 51,710 | 0.0178 | -0.092798 | ieu-b-41 | 0.831096 | 1.85E-07 | 0.002417677 | 125.3162385 |
| 58 | rs570098 | G | T | 51,710 | 0.0138 | 0.065296 | ieu-b-41 | 0.485297 | 2.23E-06 | 0.00212994 | 110.3700412 |
| 59 | rs10896090 | G | A | 51,710 | 0.0173 | -0.089704 | ieu-b-41 | 0.186671 | 2.16E-07 | 0.002443412 | 126.653421 |
| 60 | rs73496688 | A | T | 51,710 | 0.019 | 0.108702 | ieu-b-41 | 0.149117 | 1.06E-08 | 0.002998486 | 155.5120306 |
| 61 | rs12287648 | A | G | 51,710 | 0.0182 | -0.089695 | ieu-b-41 | 0.187883 | 8.29E-07 | 0.002455119 | 127.2617373 |
| 62 | rs10744560 | T | C | 51,710 | 0.014 | 0.083201 | ieu-b-41 | 0.342297 | 2.80E-09 | 0.003116879 | 161.6715125 |
| 63 | rs7969091 | G | A | 51,710 | 0.0135 | 0.0692 | ieu-b-41 | 0.438904 | 2.96E-07 | 0.002358571 | 122.2452971 |
| 64 | rs10878840 | A | G | 51,710 | 0.0138 | -0.063696 | ieu-b-41 | 0.614277 | 3.92E-06 | 0.001922623 | 99.60648967 |
| 65 | rs78781559 | A | G | 51,710 | 0.0254 | 0.117499 | ieu-b-41 | 0.0854008 | 3.73E-06 | 0.002156707 | 111.760025 |
| 66 | rs34520165 | T | C | 51,710 | 0.0156 | 0.0743 | ieu-b-41 | 0.415904 | 1.91E-06 | 0.002682162 | 139.0622032 |
| 67 | rs1819204 | A | G | 51,710 | 0.0162 | 0.080704 | ieu-b-41 | 0.736117 | 6.30E-07 | 0.002530337 | 131.1705747 |
| 68 | rs56012312 | T | C | 51,710 | 0.0142 | -0.066097 | ieu-b-41 | 0.478309 | 3.24E-06 | 0.002180296 | 112.9850686 |
| 69 | rs35955717 | C | T | 51,710 | 0.0198 | -0.094501 | ieu-b-41 | 0.145458 | 1.82E-06 | 0.002220107 | 115.0527015 |
| 70 | rs62002181 | C | G | 51,710 | 0.0191 | 0.095201 | ieu-b-41 | 0.143936 | 6.22E-07 | 0.002233514 | 115.7490693 |
| 71 | rs10147900 | T | C | 51,710 | 0.0255 | 0.1308 | ieu-b-41 | 0.140755 | 2.91E-07 | 0.004138342 | 214.8745878 |
| 72 | rs73406518 | T | C | 51,710 | 0.0235 | 0.118503 | ieu-b-41 | 0.0881358 | 4.59E-07 | 0.002257206 | 116.9796567 |
| 73 | rs325380 | C | A | 51,710 | 0.0137 | 0.064805 | ieu-b-41 | 0.56651 | 2.24E-06 | 0.002062689 | 106.8779633 |
| 74 | rs11647445 | G | T | 51,710 | 0.0142 | 0.074896 | ieu-b-41 | 0.351904 | 1.33E-07 | 0.002558649 | 132.6420298 |
| 75 | rs10492859 | G | A | 51,710 | 0.016 | -0.073297 | ieu-b-41 | 0.234522 | 4.63E-06 | 0.00192894 | 99.93439202 |
| 76 | rs66506713 | T | C | 51,710 | 0.0157 | 0.0718 | ieu-b-41 | 0.372542 | 4.80E-06 | 0.002410121 | 124.9236004 |
| 77 | rs1013191 | A | T | 51,710 | 0.0136 | -0.064699 | ieu-b-41 | 0.554096 | 1.96E-06 | 0.002068481 | 107.1787075 |
| 78 | rs8067817 | T | C | 51,710 | 0.0136 | 0.068397 | ieu-b-41 | 0.561329 | 4.93E-07 | 0.002303883 | 119.4042995 |
| 79 | rs884301 | T | C | 51,710 | 0.0138 | 0.080298 | ieu-b-41 | 0.381084 | 5.93E-09 | 0.003041528 | 157.7511557 |
| 80 | rs11557713 | A | G | 51,710 | 0.0148 | 0.0718 | ieu-b-41 | 0.291691 | 1.23E-06 | 0.002130221 | 110.3846141 |
| 81 | rs4799092 | G | C | 51,710 | 0.014 | 0.067904 | ieu-b-41 | 0.354904 | 1.23E-06 | 0.002111329 | 109.4035974 |
| 82 | rs2921552 | A | T | 51,710 | 0.016 | 0.074402 | ieu-b-41 | 0.743904 | 3.32E-06 | 0.002109206 | 109.2933225 |
| 83 | rs28821001 | C | A | 51,710 | 0.0143 | 0.066097 | ieu-b-41 | 0.358723 | 3.80E-06 | 0.002010011 | 104.142964 |
| 84 | rs2068756 | A | T | 51,710 | 0.0167 | 0.0908 | ieu-b-41 | 0.199117 | 5.41E-08 | 0.002629536 | 136.326519 |
| 85 | rs6102677 | C | T | 51,710 | 0.0148 | 0.067904 | ieu-b-41 | 0.29351 | 4.47E-06 | 0.001912272 | 99.06920027 |
| 86 | rs2143943 | T | G | 51,710 | 0.0138 | -0.065702 | ieu-b-41 | 0.560096 | 1.93E-06 | 0.002127196 | 110.227539 |
| 87 | rs1850 | C | T | 51,710 | 0.0143 | 0.075295 | ieu-b-41 | 0.353297 | 1.40E-07 | 0.00259064 | 134.3047575 |
| 88 | rs6090435 | A | G | 51,710 | 0.015 | -0.0755 | ieu-b-41 | 0.497096 | 4.82E-07 | 0.002850029 | 147.7904993 |
| 89 | rs1007893 | C | T | 51,710 | 0.0134 | 0.062003 | ieu-b-41 | 0.508904 | 3.71E-06 | 0.001921576 | 99.55217123 |
| 90 | rs5754941 | G | C | 51,710 | 0.0136 | -0.0703 | ieu-b-41 | 0.557883 | 2.35E-07 | 0.002437929 | 126.368491 |
| 91 | rs138312 | T | C | 51,710 | 0.0134 | 0.074096 | ieu-b-41 | 0.516478 | 3.21E-08 | 0.002742127 | 142.1797858 |

SNP, single nucleotide polymorphism; EA, effect allele; OA, other allele; EAF, effect allele frequency; SE, standard error; BD, bipolar disorder.

**Supplementary Table S1.6** Characteristics of instrumental variables for Depression

|  | **SNP** | **EA** | **OA** | **Samplesize** | **SE** | **β** | **id.exposure** | **EAF** | ***p* value** | **R^2^** | **F - statistic** |
| --- | --- | --- | --- | --- | --- | --- | --- | --- | --- | --- | --- |
| 1 | rs7556072 | A | G | 372,472 | 0.00757021 | -0.0378194 | finngen_R9_F5_DEPRESSIO | 0.437943 | 5.86E-07 | 0.000704137 | 262.4547422 |
| 2 | rs771124 | C | T | 372,472 | 0.0137377 | -0.0656875 | finngen_R9_F5_DEPRESSIO | 0.921477 | 1.74E-06 | 0.00062442 | 232.7230562 |
| 3 | rs6677172 | C | G | 372,472 | 0.00748747 | -0.0389446 | finngen_R9_F5_DEPRESSIO | 0.488223 | 1.98E-07 | 0.00075792 | 282.5166672 |
| 4 | rs12712502 | T | C | 372,472 | 0.0074975 | -0.0373703 | finngen_R9_F5_DEPRESSIO | 0.518463 | 6.22E-07 | 0.000697318 | 259.9111083 |
| 5 | rs35792797 | T | A | 372,472 | 0.00841312 | -0.0424795 | finngen_R9_F5_DEPRESSIO | 0.277982 | 4.44E-07 | 0.000724358 | 269.9973403 |
| 6 | rs12479064 | T | C | 372,472 | 0.0099119 | 0.0555961 | finngen_R9_F5_DEPRESSIO | 0.168975 | 2.03E-08 | 0.000868071 | 323.6112833 |
| 7 | rs72848297 | G | A | 372,472 | 0.0132281 | 0.0638356 | finngen_R9_F5_DEPRESSIO | 0.0862349 | 1.39E-06 | 0.000642205 | 239.35572 |
| 8 | rs10164593 | T | C | 372,472 | 0.0107868 | -0.0501523 | finngen_R9_F5_DEPRESSIO | 0.143296 | 3.33E-06 | 0.000617556 | 230.1633396 |
| 9 | rs67090447 | A | G | 372,472 | 0.010725 | -0.0499982 | finngen_R9_F5_DEPRESSIO | 0.143689 | 3.13E-06 | 0.000615168 | 229.2726893 |
| 10 | rs7595691 | G | A | 372,472 | 0.0162216 | -0.0747038 | finngen_R9_F5_DEPRESSIO | 0.0595314 | 4.12E-06 | 0.000624893 | 232.8994972 |
| 11 | rs6707756 | C | T | 372,472 | 0.00830476 | -0.0458768 | finngen_R9_F5_DEPRESSIO | 0.289768 | 3.31E-08 | 0.000866297 | 322.9494725 |
| 12 | rs1479551 | G | A | 372,472 | 0.00752535 | -0.0368752 | finngen_R9_F5_DEPRESSIO | 0.469685 | 9.58E-07 | 0.000677391 | 252.4788203 |
| 13 | rs259446 | C | T | 372,472 | 0.00798908 | -0.0399854 | finngen_R9_F5_DEPRESSIO | 0.675237 | 5.59E-07 | 0.000701222 | 261.3675124 |
| 14 | rs3773087 | C | T | 372,472 | 0.0108625 | -0.059413 | finngen_R9_F5_DEPRESSIO | 0.141627 | 4.51E-08 | 0.000858252 | 319.9478954 |
| 15 | rs13094224 | G | A | 372,472 | 0.00873661 | 0.0430883 | finngen_R9_F5_DEPRESSIO | 0.244305 | 8.14E-07 | 0.000685532 | 255.5152017 |
| 16 | rs530758485 | A | G | 372,472 | 0.011856 | 0.081333 | finngen_R9_F5_DEPRESSIO | 0.107383 | 6.88E-12 | 0.001268131 | 472.9406639 |
| 17 | rs4348025 | G | A | 372,472 | 0.00751699 | -0.0403294 | finngen_R9_F5_DEPRESSIO | 0.545793 | 8.09E-08 | 0.000806409 | 300.6055267 |
| 18 | rs77836957 | C | A | 372,472 | 0.018257 | -0.0878472 | finngen_R9_F5_DEPRESSIO | 0.0466757 | 1.50E-06 | 0.00068678 | 255.9805759 |
| 19 | rs192578494 | C | A | 372,472 | 0.085095 | 0.392974 | finngen_R9_F5_DEPRESSIO | 0.00173355 | 3.87E-06 | 0.000534491 | 199.1883646 |
| 20 | rs13120249 | A | G | 372,472 | 0.0137475 | 0.0628108 | finngen_R9_F5_DEPRESSIO | 0.0790525 | 4.90E-06 | 0.000574446 | 214.0868425 |
| 21 | rs113661867 | T | C | 372,472 | 0.0184234 | 0.0965125 | finngen_R9_F5_DEPRESSIO | 0.0405752 | 1.62E-07 | 0.000725218 | 270.3180884 |
| 22 | rs75540846 | T | C | 372,472 | 0.0102842 | 0.0475048 | finngen_R9_F5_DEPRESSIO | 0.155916 | 3.85E-06 | 0.000593993 | 221.3760733 |
| 23 | rs34852515 | C | T | 372,472 | 0.00819357 | -0.0380097 | finngen_R9_F5_DEPRESSIO | 0.302601 | 3.50E-06 | 0.000609776 | 227.2619667 |
| 24 | rs3792736 | T | A | 372,472 | 0.00761308 | -0.0362726 | finngen_R9_F5_DEPRESSIO | 0.587144 | 1.89E-06 | 0.000637868 | 237.7382199 |
| 25 | rs35487006 | T | G | 372,472 | 0.00765596 | -0.0360396 | finngen_R9_F5_DEPRESSIO | 0.40743 | 2.51E-06 | 0.000627166 | 233.7471604 |
| 26 | rs9293745 | A | G | 372,472 | 0.00838697 | 0.040005 | finngen_R9_F5_DEPRESSIO | 0.721922 | 1.84E-06 | 0.000642563 | 239.4891834 |
| 27 | rs147831713 | C | A | 372,472 | 0.00783273 | 0.040316 | finngen_R9_F5_DEPRESSIO | 0.349332 | 2.65E-07 | 0.000738895 | 275.4197014 |
| 28 | rs6876567 | G | A | 372,472 | 0.00763157 | 0.0508467 | finngen_R9_F5_DEPRESSIO | 0.39935 | 2.69E-11 | 0.001240311 | 462.5524691 |
| 29 | rs11743963 | T | C | 372,472 | 0.00751584 | -0.0354189 | finngen_R9_F5_DEPRESSIO | 0.479093 | 2.45E-06 | 0.000626153 | 233.3691648 |
| 30 | rs6890818 | T | C | 372,472 | 0.00952902 | 0.0465145 | finngen_R9_F5_DEPRESSIO | 0.186547 | 1.05E-06 | 0.00065664 | 244.739468 |
| 31 | rs9324959 | A | G | 372,472 | 0.00767423 | 0.0448681 | finngen_R9_F5_DEPRESSIO | 0.390377 | 5.02E-09 | 0.000958188 | 357.2387445 |
| 32 | rs190517796 | T | C | 372,472 | 0.019128 | 0.0976498 | finngen_R9_F5_DEPRESSIO | 0.0375326 | 3.31E-07 | 0.000688918 | 256.7781029 |
| 33 | rs3130160 | G | A | 372,472 | 0.00812027 | 0.0374324 | finngen_R9_F5_DEPRESSIO | 0.302063 | 4.03E-06 | 0.000590798 | 220.1846558 |
| 34 | rs9296100 | T | C | 372,472 | 0.00884014 | 0.0434263 | finngen_R9_F5_DEPRESSIO | 0.760898 | 9.00E-07 | 0.000686191 | 255.7612312 |
| 35 | rs940348 | G | A | 372,472 | 0.00888347 | 0.0477782 | finngen_R9_F5_DEPRESSIO | 0.761047 | 7.52E-08 | 0.000830259 | 309.5034932 |
| 36 | rs9491216 | G | T | 372,472 | 0.00760363 | 0.0384761 | finngen_R9_F5_DEPRESSIO | 0.574426 | 4.19E-07 | 0.000723804 | 269.7907271 |
| 37 | rs11756123 | T | A | 372,472 | 0.00775723 | -0.0539258 | finngen_R9_F5_DEPRESSIO | 0.638513 | 3.61E-12 | 0.001342411 | 500.6800753 |
| 38 | rs978164 | A | G | 372,472 | 0.00751737 | -0.0393219 | finngen_R9_F5_DEPRESSIO | 0.547975 | 1.69E-07 | 0.000765988 | 285.5264039 |
| 39 | rs144644837 | A | G | 372,472 | 0.0137101 | 0.0689059 | finngen_R9_F5_DEPRESSIO | 0.08025 | 5.01E-07 | 0.000700903 | 261.2482898 |
| 40 | rs4732435 | G | A | 372,472 | 0.00786268 | 0.0397918 | finngen_R9_F5_DEPRESSIO | 0.634262 | 4.17E-07 | 0.000734608 | 273.8207759 |
| 41 | rs11979413 | C | A | 372,472 | 0.00992858 | 0.0459539 | finngen_R9_F5_DEPRESSIO | 0.822392 | 3.68E-06 | 0.000616902 | 229.9193086 |
| 42 | rs10088768 | C | G | 372,472 | 0.0093036 | 0.0425151 | finngen_R9_F5_DEPRESSIO | 0.199435 | 4.88E-06 | 0.000577184 | 215.1079302 |
| 43 | rs34115597 | G | T | 372,472 | 0.00813691 | -0.0373173 | finngen_R9_F5_DEPRESSIO | 0.30798 | 4.51E-06 | 0.000593597 | 221.2283375 |
| 44 | rs7842714 | A | T | 372,472 | 0.00774148 | 0.0365288 | finngen_R9_F5_DEPRESSIO | 0.600865 | 2.37E-06 | 0.000640026 | 238.5431085 |
| 45 | rs13249798 | G | C | 372,472 | 0.0125246 | -0.0602644 | finngen_R9_F5_DEPRESSIO | 0.101453 | 1.50E-06 | 0.000662151 | 246.7949819 |
| 46 | rs57268239 | A | C | 372,472 | 0.00926038 | 0.0510729 | finngen_R9_F5_DEPRESSIO | 0.203937 | 3.48E-08 | 0.000846944 | 315.7284922 |
| 47 | rs10990411 | C | T | 372,472 | 0.0113176 | -0.0543872 | finngen_R9_F5_DEPRESSIO | 0.131207 | 1.54E-06 | 0.000674368 | 251.3512143 |
| 48 | rs10759925 | C | T | 372,472 | 0.00786224 | -0.0375299 | finngen_R9_F5_DEPRESSIO | 0.351599 | 1.81E-06 | 0.000642209 | 239.3571546 |
| 49 | rs12763632 | G | A | 372,472 | 0.00906568 | 0.0415112 | finngen_R9_F5_DEPRESSIO | 0.215025 | 4.67E-06 | 0.000581708 | 216.7950485 |
| 50 | rs36090777 | C | G | 372,472 | 0.0122893 | -0.0597606 | finngen_R9_F5_DEPRESSIO | 0.108564 | 1.16E-06 | 0.000691251 | 257.6484348 |
| 51 | rs72481178 | C | T | 372,472 | 0.0185334 | -0.0880699 | finngen_R9_F5_DEPRESSIO | 0.0452558 | 2.01E-06 | 0.000670265 | 249.8208985 |
| 52 | rs1027190 | G | T | 372,472 | 0.00834093 | -0.0459014 | finngen_R9_F5_DEPRESSIO | 0.722341 | 3.73E-08 | 0.000845154 | 315.0608065 |
| 53 | rs117763266 | T | C | 372,472 | 0.0221313 | -0.101108 | finngen_R9_F5_DEPRESSIO | 0.0308913 | 4.91E-06 | 0.000612082 | 228.1218668 |
| 54 | rs927456 | C | T | 372,472 | 0.00771541 | 0.0365299 | finngen_R9_F5_DEPRESSIO | 0.376953 | 2.19E-06 | 0.000626809 | 233.6138461 |
| 55 | rs12804093 | C | T | 372,472 | 0.00793574 | 0.038152 | finngen_R9_F5_DEPRESSIO | 0.327943 | 1.53E-06 | 0.000641607 | 239.1327859 |
| 56 | rs587925 | G | A | 372,472 | 0.00776064 | -0.0402559 | finngen_R9_F5_DEPRESSIO | 0.63686 | 2.13E-07 | 0.000749561 | 279.3985127 |
| 57 | rs2187987 | T | G | 372,472 | 0.00821121 | 0.0407482 | finngen_R9_F5_DEPRESSIO | 0.291282 | 6.96E-07 | 0.000685542 | 255.5189375 |
| 58 | rs534804 | A | G | 372,472 | 0.00922321 | 0.0447515 | finngen_R9_F5_DEPRESSIO | 0.208091 | 1.22E-06 | 0.000660045 | 246.0094629 |
| 59 | rs4882348 | T | C | 372,472 | 0.00750864 | -0.0381598 | finngen_R9_F5_DEPRESSIO | 0.538341 | 3.73E-07 | 0.000723804 | 269.7905265 |
| 60 | rs1995514 | A | G | 372,472 | 0.00769068 | -0.0416291 | finngen_R9_F5_DEPRESSIO | 0.391963 | 6.20E-08 | 0.000826036 | 307.9280913 |
| 61 | rs11109659 | C | A | 372,472 | 0.0130426 | 0.0601382 | finngen_R9_F5_DEPRESSIO | 0.088503 | 4.01E-06 | 0.000583504 | 217.4647487 |
| 62 | rs10507576 | A | G | 372,472 | 0.0188169 | 0.0949104 | finngen_R9_F5_DEPRESSIO | 0.040122 | 4.56E-07 | 0.000693835 | 258.6121631 |
| 63 | rs9570498 | A | C | 372,472 | 0.0153391 | -0.070633 | finngen_R9_F5_DEPRESSIO | 0.0669041 | 4.13E-06 | 0.000622909 | 232.1593738 |
| 64 | rs142330459 | A | G | 372,472 | 0.0258578 | 0.120758 | finngen_R9_F5_DEPRESSIO | 0.0202231 | 3.01E-06 | 0.000577879 | 215.3669637 |
| 65 | rs17117590 | G | A | 372,472 | 0.0209482 | 0.0998261 | finngen_R9_F5_DEPRESSIO | 0.031911 | 1.88E-06 | 0.000615707 | 229.4735759 |
| 66 | rs11159917 | T | C | 372,472 | 0.00866968 | 0.0402987 | finngen_R9_F5_DEPRESSIO | 0.243837 | 3.35E-06 | 0.000598862 | 223.1919658 |
| 67 | rs113909596 | A | G | 372,472 | 0.0214016 | 0.105733 | finngen_R9_F5_DEPRESSIO | 0.0324778 | 7.80E-07 | 0.000702585 | 261.8756884 |
| 68 | rs1036810 | C | G | 372,472 | 0.00753061 | -0.0355139 | finngen_R9_F5_DEPRESSIO | 0.545513 | 2.41E-06 | 0.000625393 | 233.0860539 |
| 69 | rs215896 | C | T | 372,472 | 0.00863932 | 0.0519425 | finngen_R9_F5_DEPRESSIO | 0.248004 | 1.83E-09 | 0.001006352 | 375.2135216 |
| 70 | rs7184244 | A | C | 372,472 | 0.00755322 | 0.0357005 | finngen_R9_F5_DEPRESSIO | 0.470113 | 2.28E-06 | 0.000634986 | 236.6634962 |
| 71 | rs57852066 | A | G | 372,472 | 0.0168294 | 0.0941695 | finngen_R9_F5_DEPRESSIO | 0.0492689 | 2.20E-08 | 0.000830771 | 309.6943908 |
| 72 | rs9916184 | T | G | 372,472 | 0.0114653 | 0.0612116 | finngen_R9_F5_DEPRESSIO | 0.128576 | 9.35E-08 | 0.000839628 | 312.9990246 |
| 73 | rs9890900 | C | T | 372,472 | 0.0123265 | 0.0630472 | finngen_R9_F5_DEPRESSIO | 0.101217 | 3.14E-07 | 0.000723219 | 269.5723945 |
| 74 | rs12602745 | C | T | 372,472 | 0.00901495 | -0.0412079 | finngen_R9_F5_DEPRESSIO | 0.222688 | 4.85E-06 | 0.000587873 | 219.0936712 |
| 75 | rs140005264 | T | G | 372,472 | 0.0162097 | 0.0806647 | finngen_R9_F5_DEPRESSIO | 0.0539694 | 6.48E-07 | 0.000664431 | 247.645117 |
| 76 | rs113823870 | A | G | 372,472 | 0.0151251 | -0.0785054 | finngen_R9_F5_DEPRESSIO | 0.0681958 | 2.10E-07 | 0.00078327 | 291.9731741 |

SNP, single nucleotide polymorphism; EA, effect allele; OA, other allele; EAF, effect allele frequency; SE, standard error.

**Supplementary Table S1.7** Characteristics of instrumental variables for OCD

|  | **SNP** | **EA** | **OA** | **Samplesize** | **SE** | **β** | **id.exposure** | **EAF** | ***p* value** | **R^2^** | **F - statistic** |
| --- | --- | --- | --- | --- | --- | --- | --- | --- | --- | --- | --- |
| 1 | rs13427136 | C | T | 339,539 | 0.052694 | 0.273179 | finngen_R9_F5_OCD | 0.0940931 | 2.17E-07 | 0.01272231 | 4375.359534 |
| 2 | rs11717238 | G | A | 339,539 | 0.032662 | -0.149474 | finngen_R9_F5_OCD | 0.508754 | 4.73E-06 | 0.011167814 | 3834.711412 |
| 3 | rs315678 | T | C | 339,539 | 0.0565215 | 0.269177 | finngen_R9_F5_OCD | 0.894216 | 1.91E-06 | 0.013707818 | 4718.998402 |
| 4 | rs79239243 | A | C | 339,539 | 0.0979061 | 0.453007 | finngen_R9_F5_OCD | 0.0214003 | 3.71E-06 | 0.008595374 | 2943.750019 |
| 5 | rs12213997 | C | T | 339,539 | 0.0434961 | 0.198928 | finngen_R9_F5_OCD | 0.152532 | 4.80E-06 | 0.010230718 | 3509.613058 |
| 6 | rs80216287 | T | G | 339,539 | 0.0452259 | 0.216118 | finngen_R9_F5_OCD | 0.135043 | 1.76E-06 | 0.01091135 | 3745.6773 |
| 7 | rs60562275 | T | C | 339,539 | 0.0518566 | 0.238119 | finngen_R9_F5_OCD | 0.098297 | 4.39E-06 | 0.010051292 | 3447.436596 |
| 8 | rs118191920 | A | G | 339,539 | 0.0788855 | -0.389995 | finngen_R9_F5_OCD | 0.0569597 | 7.66E-07 | 0.016339773 | 5640.115724 |
| 9 | rs58466321 | C | T | 339,539 | 0.0570772 | 0.272631 | finngen_R9_F5_OCD | 0.0770307 | 1.78E-06 | 0.010568943 | 3626.879593 |
| 10 | rs17466747 | A | G | 339,539 | 0.0348176 | 0.175437 | finngen_R9_F5_OCD | 0.292637 | 4.69E-07 | 0.012742186 | 4382.283635 |
| 11 | rs76110959 | G | A | 339,539 | 0.192575 | -0.882499 | finngen_R9_F5_OCD | 0.0120103 | 4.59E-06 | 0.01848267 | 6393.723444 |
| 12 | rs74924645 | A | G | 339,539 | 0.0562867 | -0.266287 | finngen_R9_F5_OCD | 0.104603 | 2.24E-06 | 0.013282802 | 4570.714659 |
| 13 | rs7207457 | T | C | 339,539 | 0.0356018 | -0.170155 | finngen_R9_F5_OCD | 0.321654 | 1.76E-06 | 0.012634546 | 4344.790404 |
| 14 | rs55888334 | A | G | 339,539 | 0.0627743 | 0.315626 | finngen_R9_F5_OCD | 0.0603796 | 4.96E-07 | 0.011303637 | 3881.88243 |
| 15 | rs8084719 | G | C | 339,539 | 0.0370263 | -0.185983 | finngen_R9_F5_OCD | 0.281141 | 5.09E-07 | 0.013981198 | 4814.445821 |
| 16 | rs6105964 | A | G | 339,539 | 0.0537313 | 0.245975 | finngen_R9_F5_OCD | 0.088318 | 4.70E-06 | 0.009743266 | 3340.748966 |

SNP, single nucleotide polymorphism; EA, effect allele; OA, other allele; EAF, effect allele frequency; SE, standard error; OCD, obsessive compulsive disorder.

**Supplementary Table S1.8** Characteristics of instrumental variables for SCZ

|  | **SNP** | **EA** | **OA** | **Samplesize** | **SE** | **β** | **id.exposure** | **EAF** | ***p* value** | **R^2^** | **F - statistic** |
| --- | --- | --- | --- | --- | --- | --- | --- | --- | --- | --- | --- |
| 1 | rs12652777 | C | T | 370,675 | 0.0262846 | -0.125906 | finngen_R9_F5_SCHZPHR | 0.531128 | 1.67E-06 | 0.00789544 | 2949.917375 |
| 2 | rs9367849 | G | C | 370,675 | 0.0414786 | 0.206682 | finngen_R9_F5_SCHZPHR | 0.110842 | 6.26E-07 | 0.008420126 | 3147.616766 |
| 3 | rs6966281 | T | G | 370,675 | 0.027283 | 0.13287 | finngen_R9_F5_SCHZPHR | 0.62431 | 1.12E-06 | 0.008281591 | 3095.397093 |
| 4 | rs80141503 | A | G | 370,675 | 0.0276429 | 0.126344 | finngen_R9_F5_SCHZPHR | 0.647207 | 4.86E-06 | 0.007289578 | 2721.8913 |
| 5 | rs77012313 | C | A | 370,675 | 0.0993477 | -0.459696 | finngen_R9_F5_SCHZPHR | 0.0206273 | 3.71E-06 | 0.008538112 | 3192.101942 |
| 6 | rs2675600 | A | G | 370,675 | 0.0391568 | -0.180013 | finngen_R9_F5_SCHZPHR | 0.880767 | 4.28E-06 | 0.006806052 | 2540.107688 |
| 7 | rs1641714 | A | G | 370,675 | 0.0299733 | 0.143081 | finngen_R9_F5_SCHZPHR | 0.250363 | 1.81E-06 | 0.007684491 | 2870.491492 |
| 8 | rs59665366 | A | C | 370,675 | 0.0341188 | 0.156932 | finngen_R9_F5_SCHZPHR | 0.170414 | 4.23E-06 | 0.006963374 | 2599.234038 |
| 9 | rs58968563 | T | C | 370,675 | 0.0549836 | -0.276263 | finngen_R9_F5_SCHZPHR | 0.0666519 | 5.05E-07 | 0.009495801 | 3553.581308 |
| 10 | rs72827147 | G | T | 370,675 | 0.0590435 | -0.28163 | finngen_R9_F5_SCHZPHR | 0.0564573 | 1.84E-06 | 0.008450249 | 3158.973136 |
| 11 | rs144031898 | T | C | 370,675 | 0.0776489 | -0.358446 | finngen_R9_F5_SCHZPHR | 0.0322003 | 3.91E-06 | 0.008007978 | 2992.303544 |
| 12 | rs62203513 | A | G | 370,675 | 0.0354067 | -0.162663 | finngen_R9_F5_SCHZPHR | 0.169284 | 4.35E-06 | 0.007441766 | 2779.14365 |

SNP, single nucleotide polymorphism; EA, effect allele; OA, other allele; EAF, effect allele frequency; SE, standard error; SCZ, schizophrenia.

**Supplementary Table S2.1** SNPs from GWAS on ADHD and CVD

|  |  | | | **Exposure (ADHD)** | | |  | **Outcome (CVD)** | | | | |
| --- | --- | --- | --- | --- | --- | --- | --- | --- | --- | --- | --- | --- |
|  | **SNP** | **EA** | **OA** | **β** | **SE** | ***p* value** |  | **Case** | **Control** | **β** | **SE** | ***p* value** |
| 1 | rs10262192 | A | G | 0.073204 | 0.0132 | 2.93E-08 |  | 185,353 | 191,924 | 0.00252645 | 0.00567629 | 0.656256 |
| 2 | rs10400180 | G | T | -0.0925974 | 0.0163 | 1.34E-08 |  | 185,353 | 191,924 | -0.0113563 | 0.00644113 | 0.0778861 |
| 3 | rs10444728 | A | G | -0.0679042 | 0.0139 | 1.03E-06 |  | 185,353 | 191,924 | -0.00752414 | 0.00561678 | 0.180381 |
| 4 | rs1077612 | T | C | -0.0769026 | 0.0165 | 3.15E-06 |  | 185,353 | 191,924 | 0.00335874 | 0.00642376 | 0.60107 |
| 5 | rs10965173 | G | A | -0.0783027 | 0.0167 | 2.75E-06 |  | 185,353 | 191,924 | -0.00251872 | 0.00656873 | 0.701392 |
| 6 | rs11698378 | C | T | 0.0827948 | 0.0175 | 2.23E-06 |  | 185,353 | 191,924 | -0.000747625 | 0.00747795 | 0.920362 |
| 7 | rs143912172 | C | A | -0.138796 | 0.029 | 1.70E-06 |  | 185,353 | 191,924 | -0.000674992 | 0.01128 | 0.952283 |
| 8 | rs1484144 | C | T | -0.0608975 | 0.0133 | 4.68E-06 |  | 185,353 | 191,924 | 0.00848904 | 0.00561458 | 0.130543 |
| 9 | rs1513155 | A | G | -0.0849045 | 0.0182 | 3.08E-06 |  | 185,353 | 191,924 | 0.0160336 | 0.00785821 | 0.0413143 |
| 10 | rs17531412 | G | A | -0.105602 | 0.0145 | 3.27E-13 |  | 185,353 | 191,924 | 0.0101388 | 0.00590059 | 0.0857472 |
| 11 | rs180822580 | A | G | -0.155298 | 0.0324 | 1.64E-06 |  | 185,353 | 191,924 | 0.00039074 | 0.0109685 | 0.971582 |
| 12 | rs212178 | A | G | -0.1154 | 0.02 | 7.93E-09 |  | 185,353 | 191,924 | -0.00826428 | 0.0115366 | 0.473773 |
| 13 | rs2144782 | T | C | 0.074003 | 0.0143 | 2.28E-07 |  | 185,353 | 191,924 | 0.017592 | 0.00588569 | 0.00279943 |
| 14 | rs2243517 | C | T | 0.072001 | 0.0136 | 1.20E-07 |  | 185,353 | 191,924 | -0.00230883 | 0.00569929 | 0.685398 |
| 15 | rs227378 | A | C | 0.0740958 | 0.0143 | 2.20E-07 |  | 185,353 | 191,924 | 0.00897773 | 0.00592321 | 0.129599 |
| 16 | rs2835344 | T | C | -0.0754997 | 0.0158 | 1.77E-06 |  | 185,353 | 191,924 | 0.00742355 | 0.00612561 | 0.225555 |
| 17 | rs4144756 | A | G | 0.0763961 | 0.0145 | 1.37E-07 |  | 185,353 | 191,924 | -0.00336878 | 0.00578897 | 0.560613 |
| 18 | rs433274 | C | T | 0.0934978 | 0.0199 | 2.62E-06 |  | 185,353 | 191,924 | 0.0146266 | 0.00992501 | 0.140558 |
| 19 | rs4839923 | A | G | 0.0666956 | 0.0133 | 5.31E-07 |  | 185,353 | 191,924 | 0.00673726 | 0.00558846 | 0.227985 |
| 20 | rs60798171 | G | T | 0.0733021 | 0.0155 | 2.25E-06 |  | 185,353 | 191,924 | 0.00779139 | 0.00675325 | 0.248613 |
| 21 | rs61896068 | A | G | 0.0744022 | 0.0161 | 3.81E-06 |  | 185,353 | 191,924 | 0.00985599 | 0.00654804 | 0.132277 |
| 22 | rs62259516 | T | C | 0.143104 | 0.0286 | 5.63E-07 |  | 185,353 | 191,924 | 0.00300853 | 0.0118861 | 0.800181 |
| 23 | rs6933023 | T | C | 0.066602 | 0.0133 | 5.51E-07 |  | 185,353 | 191,924 | 0.000105084 | 0.00570625 | 0.985307 |
| 24 | rs74760947 | G | A | 0.179797 | 0.0317 | 1.41E-08 |  | 185,353 | 191,924 | 0.00245839 | 0.0111156 | 0.824963 |
| 25 | rs7631360 | G | A | -0.0654974 | 0.0137 | 1.75E-06 |  | 185,353 | 191,924 | 0.000964143 | 0.0058287 | 0.868619 |
| 26 | rs7634587 | G | A | -0.0635005 | 0.0137 | 3.57E-06 |  | 185,353 | 191,924 | -0.00918331 | 0.0060958 | 0.13194 |
| 27 | rs7989860 | A | G | 0.0639978 | 0.0132 | 1.25E-06 |  | 185,353 | 191,924 | 0.00299835 | 0.00560878 | 0.592939 |
| 28 | rs8058677 | T | C | 0.0680978 | 0.0135 | 4.55E-07 |  | 185,353 | 191,924 | 0.00504178 | 0.0056133 | 0.369086 |
| 29 | rs9661242 | G | A | 0.0625991 | 0.0135 | 3.54E-06 |  | 185,353 | 191,924 | -0.000459096 | 0.00562978 | 0.935006 |
| 30 | rs9677504 | A | G | 0.116903 | 0.0206 | 1.39E-08 |  | 185,353 | 191,924 | 0.00115301 | 0.00904587 | 0.898574 |
| 31 | rs992936 | C | T | -0.0731018 | 0.0135 | 6.13E-08 |  | 185,353 | 191,924 | 0.000626751 | 0.00563916 | 0.911503 |

SNP, single nucleotide polymorphism; EA, effect allele; OA, other allele; SE, standard error; ADHD, attention deficit hyperactivity disorder; CVD, cardiovascular diseases.

**Supplementary Table S2.2** SNPs from GWAS on AN and CVD

|  |  | | | **Exposure (AN)** | | |  | **Outcome (CVD)** | | | | |
| --- | --- | --- | --- | --- | --- | --- | --- | --- | --- | --- | --- | --- |
|  | **SNP** | **EA** | **OA** | **β** | **SE** | ***p* value** |  | **Case** | **Control** | **β** | **SE** | ***p* value** |
| 1 | rs111925923 | T | C | 0.409497 | 0.089 | 4.20E-06 |  | 185,353 | 191,924 | 0.0119344 | 0.0160606 | 0.457428 |
| 2 | rs117957029 | C | T | 0.536502 | 0.1024 | 1.61E-07 |  | 185,353 | 191,924 | -0.00424874 | 0.0187542 | 0.820775 |
| 3 | rs13125782 | C | T | -0.174802 | 0.0356 | 9.10E-07 |  | 185,353 | 191,924 | 6.67E-06 | 0.00670508 | 0.999206 |
| 4 | rs145106667 | G | A | 0.686291 | 0.1483 | 3.70E-06 |  | 185,353 | 191,924 | -0.0230409 | 0.0266959 | 0.38809 |
| 5 | rs1894792 | T | C | 0.172599 | 0.0364 | 2.12E-06 |  | 185,353 | 191,924 | 0.00636762 | 0.00633263 | 0.314644 |
| 6 | rs2345557 | T | G | 0.237299 | 0.051 | 3.27E-06 |  | 185,353 | 191,924 | -0.0176026 | 0.0113473 | 0.120839 |
| 7 | rs4763166 | G | A | -0.152403 | 0.0298 | 3.15E-07 |  | 185,353 | 191,924 | 0.00184121 | 0.00564572 | 0.744331 |
| 8 | rs75115477 | G | A | 0.277204 | 0.0593 | 2.95E-06 |  | 185,353 | 191,924 | -0.0129491 | 0.013536 | 0.338745 |
| 9 | rs75307524 | G | A | 0.474397 | 0.101 | 2.64E-06 |  | 185,353 | 191,924 | -0.0184206 | 0.0183965 | 0.316676 |
| 10 | rs75544652 | T | C | 0.5223 | 0.112 | 3.11E-06 |  | 185,353 | 191,924 | 0.0937633 | 0.0696135 | 0.178009 |
| 11 | rs79350580 | T | C | 0.309196 | 0.0666 | 3.44E-06 |  | 185,353 | 191,924 | 0.00881599 | 0.016869 | 0.601242 |

SNP, single nucleotide polymorphism; EA, effect allele; OA, other allele; SE, standard error; AN, anorexia nervosa; CVD, cardiovascular diseases.

**Supplementary Table S2.3** SNPs from GWAS on ANX and CVD

|  |  | | | **Exposure (ANX)** | | |  | **Outcome (CVD)** | | | | |
| --- | --- | --- | --- | --- | --- | --- | --- | --- | --- | --- | --- | --- |
|  | **SNP** | **EA** | **OA** | **β** | **SE** | ***p* value** |  | **Case** | **Control** | **β** | **SE** | ***p* value** |
| 1 | rs10078559 | G | T | 0.149171 | 0.0294875 | 4.22E-07 |  | 185,353 | 191,924 | 0.0103872 | 0.0175717 | 0.55443 |
| 2 | rs1059307 | T | G | 0.0463918 | 0.00968404 | 1.66E-06 |  | 185,353 | 191,924 | 0.0033033 | 0.00559199 | 0.554707 |
| 3 | rs1119988 | C | T | -0.0487858 | 0.00986764 | 7.65E-07 |  | 185,353 | 191,924 | -0.00626957 | 0.00567441 | 0.269209 |
| 4 | rs11243619 | T | G | -0.0592033 | 0.0123952 | 1.79E-06 |  | 185,353 | 191,924 | -0.0184199 | 0.00708152 | 0.0092918 |
| 5 | rs117632798 | T | A | -0.101036 | 0.0193497 | 1.77E-07 |  | 185,353 | 191,924 | -0.00336812 | 0.0110036 | 0.759535 |
| 6 | rs11857461 | T | C | 0.0469825 | 0.00968185 | 1.22E-06 |  | 185,353 | 191,924 | 0.00153918 | 0.00559307 | 0.783166 |
| 7 | rs12498967 | A | G | 0.052111 | 0.010545 | 7.74E-07 |  | 185,353 | 191,924 | 0.00588651 | 0.00612904 | 0.336839 |
| 8 | rs12614303 | T | C | -0.0476777 | 0.00972451 | 9.45E-07 |  | 185,353 | 191,924 | -0.00467267 | 0.00560483 | 0.404458 |
| 9 | rs13244325 | G | T | -0.0636689 | 0.013909 | 4.70E-06 |  | 185,353 | 191,924 | -0.00537855 | 0.00794787 | 0.498578 |
| 10 | rs145525394 | C | T | -0.0963933 | 0.0185721 | 2.10E-07 |  | 185,353 | 191,924 | -0.0129277 | 0.0104613 | 0.216546 |
| 11 | rs147631399 | G | A | 0.28299 | 0.0586524 | 1.40E-06 |  | 185,353 | 191,924 | 0.0167006 | 0.0358695 | 0.641506 |
| 12 | rs189295234 | A | G | 0.219074 | 0.0475714 | 4.12E-06 |  | 185,353 | 191,924 | -0.0168121 | 0.0284556 | 0.55464 |
| 13 | rs2051523 | A | G | 0.0688427 | 0.0132834 | 2.19E-07 |  | 185,353 | 191,924 | 0.0050309 | 0.00776928 | 0.517284 |
| 14 | rs215894 | A | T | 0.0510017 | 0.0100136 | 3.52E-07 |  | 185,353 | 191,924 | 0.00449743 | 0.00579692 | 0.43785 |
| 15 | rs2281530 | C | T | -0.0447306 | 0.00975356 | 4.52E-06 |  | 185,353 | 191,924 | -0.0154833 | 0.00562403 | 0.00590419 |
| 16 | rs2397085 | C | T | -0.0874888 | 0.018698 | 2.88E-06 |  | 185,353 | 191,924 | -0.0188905 | 0.0107054 | 0.0776354 |
| 17 | rs250839 | G | T | 0.05162 | 0.0106807 | 1.34E-06 |  | 185,353 | 191,924 | 0.0133205 | 0.00613591 | 0.0299378 |
| 18 | rs2756119 | A | G | -0.0507645 | 0.0100408 | 4.29E-07 |  | 185,353 | 191,924 | -0.00105775 | 0.00577019 | 0.854553 |
| 19 | rs28478424 | G | T | 0.0784687 | 0.0151606 | 2.27E-07 |  | 185,353 | 191,924 | 0.000718701 | 0.00888164 | 0.935506 |
| 20 | rs34832985 | G | A | -0.054433 | 0.0099834 | 4.97E-08 |  | 185,353 | 191,924 | -0.0013148 | 0.00577828 | 0.820002 |
| 21 | rs4330896 | A | G | 0.0563465 | 0.0107951 | 1.79E-07 |  | 185,353 | 191,924 | 0.00336845 | 0.00619222 | 0.586454 |
| 22 | rs4772087 | T | C | 0.0575959 | 0.00983936 | 4.81E-09 |  | 185,353 | 191,924 | 0.0044095 | 0.00570009 | 0.439176 |
| 23 | rs55945133 | T | C | 0.0878536 | 0.0176946 | 6.87E-07 |  | 185,353 | 191,924 | -0.00693893 | 0.010407 | 0.504926 |
| 24 | rs56258708 | G | A | 0.0558644 | 0.0119959 | 3.21E-06 |  | 185,353 | 191,924 | 0.0100646 | 0.00697608 | 0.149095 |
| 25 | rs57852066 | A | G | 0.103968 | 0.0217339 | 1.72E-06 |  | 185,353 | 191,924 | 0.0145711 | 0.0129586 | 0.260828 |
| 26 | rs61820769 | T | C | -0.13383 | 0.026501 | 4.42E-07 |  | 185,353 | 191,924 | -0.0418353 | 0.0148091 | 0.00472846 |
| 27 | rs62126622 | G | T | 0.220803 | 0.0469004 | 2.50E-06 |  | 185,353 | 191,924 | 0.00766108 | 0.0284468 | 0.787689 |
| 28 | rs6450476 | G | A | 0.0564773 | 0.0107966 | 1.69E-07 |  | 185,353 | 191,924 | 0.00773571 | 0.00620037 | 0.212171 |
| 29 | rs6751342 | C | A | -0.0481313 | 0.0101762 | 2.25E-06 |  | 185,353 | 191,924 | -0.00607255 | 0.00586245 | 0.300278 |
| 30 | rs6965423 | C | T | 0.045814 | 0.00968997 | 2.27E-06 |  | 185,353 | 191,924 | 0.0097585 | 0.00559184 | 0.0809618 |
| 31 | rs72723168 | T | C | 0.117612 | 0.0255836 | 4.28E-06 |  | 185,353 | 191,924 | 0.0104604 | 0.0152016 | 0.491381 |
| 32 | rs72805914 | A | C | 0.152923 | 0.0304827 | 5.26E-07 |  | 185,353 | 191,924 | 0.00448739 | 0.0182604 | 0.80588 |
| 33 | rs72967615 | T | G | 0.114426 | 0.0247645 | 3.83E-06 |  | 185,353 | 191,924 | -0.0199125 | 0.0146279 | 0.17343 |
| 34 | rs7379967 | A | G | 0.0454037 | 0.00973847 | 3.13E-06 |  | 185,353 | 191,924 | 0.00565427 | 0.00562088 | 0.314444 |
| 35 | rs7555230 | C | T | 0.0466512 | 0.0101682 | 4.48E-06 |  | 185,353 | 191,924 | -0.000979643 | 0.00590219 | 0.868173 |
| 36 | rs7570682 | A | G | 0.0555894 | 0.0112745 | 8.20E-07 |  | 185,353 | 191,924 | 0.017793 | 0.0065691 | 0.00675709 |
| 37 | rs78175056 | C | T | 0.0506886 | 0.0100688 | 4.80E-07 |  | 185,353 | 191,924 | 0.00475626 | 0.00583522 | 0.415018 |
| 38 | rs79634932 | G | C | 0.0678968 | 0.0138332 | 9.19E-07 |  | 185,353 | 191,924 | 0.00826918 | 0.00806358 | 0.305129 |
| 39 | rs869208 | A | G | -0.056345 | 0.0120081 | 2.70E-06 |  | 185,353 | 191,924 | -0.000270481 | 0.00684498 | 0.96848 |

SNP, single nucleotide polymorphism; EA, effect allele; OA, other allele; SE, standard error; ANX, anxiety disorder; CVD, cardiovascular diseases.

**Supplementary Table S2.4** SNPs from GWAS on ASD and CVD

|  |  | | | **Exposure (ASD)** | | |  | **Outcome (CVD)** | | | | |
| --- | --- | --- | --- | --- | --- | --- | --- | --- | --- | --- | --- | --- |
|  | **SNP** | **EA** | **OA** | **β** | **SE** | ***p* value** |  | **Case** | **Control** | **β** | **SE** | ***p* value** |
| 1 | rs11185408 | A | G | -0.0686965 | 0.0138 | 6.42E-07 |  | 185,353 | 191,924 | 0.00324714 | 0.00559452 | 0.561636 |
| 2 | rs144911765 | C | T | 0.190096 | 0.0403 | 2.39E-06 |  | 185,353 | 191,924 | -0.000523972 | 0.0127006 | 0.967092 |
| 3 | rs149923766 | G | T | 0.237306 | 0.0484 | 9.44E-07 |  | 185,353 | 191,924 | -0.0218943 | 0.0186146 | 0.239519 |
| 4 | rs16879023 | A | G | -0.0957953 | 0.0201 | 1.88E-06 |  | 185,353 | 191,924 | -0.0187129 | 0.00874245 | 0.0323177 |
| 5 | rs2224274 | T | C | 0.0709989 | 0.0138 | 2.68E-07 |  | 185,353 | 191,924 | 0.0112611 | 0.00562856 | 0.0454234 |
| 6 | rs2391769 | G | A | 0.0769026 | 0.0145 | 1.14E-07 |  | 185,353 | 191,924 | 0.00757322 | 0.00610964 | 0.215141 |
| 7 | rs2635182 | T | C | 0.0669014 | 0.014 | 1.76E-06 |  | 185,353 | 191,924 | 0.0168045 | 0.00571153 | 0.00325874 |
| 8 | rs28729902 | G | A | 0.0839035 | 0.0178 | 2.43E-06 |  | 185,353 | 191,924 | -0.0038685 | 0.00674562 | 0.566318 |
| 9 | rs292441 | A | G | -0.0724954 | 0.0149 | 1.14E-06 |  | 185,353 | 191,924 | -0.0164684 | 0.0062587 | 0.00850629 |
| 10 | rs35404050 | T | C | 0.0843044 | 0.0176 | 1.67E-06 |  | 185,353 | 191,924 | 0.00399661 | 0.00725691 | 0.581818 |
| 11 | rs45595836 | T | C | 0.138996 | 0.0272 | 3.22E-07 |  | 185,353 | 191,924 | 0.0164688 | 0.0127261 | 0.195632 |
| 12 | rs4750990 | C | T | 0.0680968 | 0.0141 | 1.37E-06 |  | 185,353 | 191,924 | 0.00665241 | 0.00565289 | 0.239269 |
| 13 | rs644552 | A | G | 0.159403 | 0.0346 | 4.08E-06 |  | 185,353 | 191,924 | 0.0143635 | 0.0136987 | 0.294393 |
| 14 | rs6692705 | G | A | -0.0656005 | 0.0141 | 3.28E-06 |  | 185,353 | 191,924 | -0.000461039 | 0.00579055 | 0.93654 |
| 15 | rs76397219 | G | A | 0.140297 | 0.0303 | 3.65E-06 |  | 185,353 | 191,924 | -0.0086118 | 0.0134644 | 0.522435 |
| 16 | rs77691144 | C | T | 0.207406 | 0.0435 | 1.86E-06 |  | 185,353 | 191,924 | 0.0158463 | 0.0256406 | 0.536563 |
| 17 | rs7783557 | C | T | -0.0670042 | 0.0146 | 4.45E-06 |  | 185,353 | 191,924 | 0.000653221 | 0.00592848 | 0.912264 |
| 18 | rs78653484 | T | C | -0.176296 | 0.0385 | 4.67E-06 |  | 185,353 | 191,924 | 0.00973329 | 0.0119647 | 0.41593 |
| 19 | rs78827416 | A | G | 0.130502 | 0.0266 | 9.29E-07 |  | 185,353 | 191,924 | 0.000610768 | 0.00914404 | 0.946746 |
| 20 | rs79940520 | G | A | 0.0953992 | 0.0207 | 4.05E-06 |  | 185,353 | 191,924 | 0.00572619 | 0.00877438 | 0.514012 |
| 21 | rs9366877 | G | A | -0.0684994 | 0.0139 | 8.31E-07 |  | 185,353 | 191,924 | -0.00933197 | 0.00581512 | 0.108543 |
| 22 | rs9389208 | T | C | 0.0672006 | 0.0144 | 3.06E-06 |  | 185,353 | 191,924 | -0.0075128 | 0.00565873 | 0.184295 |

SNP, single nucleotide polymorphism; EA, effect allele; OA, other allele; SE, standard error; ASD, autism spectrum disorder; CVD, cardiovascular diseases.

**Supplementary Table S2.5** SNPs from GWAS on BD and CVD

|  |  | | | **Exposure (BD)** | | |  | **Outcome (CVD)** | | | | |
| --- | --- | --- | --- | --- | --- | --- | --- | --- | --- | --- | --- | --- |
|  | **SNP** | **EA** | **OA** | **β** | **SE** | ***p* value** |  | **Case** | **Control** | **β** | **SE** | ***p* value** |
| 1 | rs10075788 | G | A | 0.071195 | 0.0143 | 6.40E-07 |  | 185,353 | 191,924 | 0.00517372 | 0.00601617 | 0.389806 |
| 2 | rs1007893 | C | T | 0.062003 | 0.0134 | 3.71E-06 |  | 185,353 | 191,924 | 0.00714432 | 0.00561391 | 0.203157 |
| 3 | rs10092482 | T | C | 0.083799 | 0.0172 | 1.10E-06 |  | 185,353 | 191,924 | -0.00882132 | 0.0074464 | 0.236159 |
| 4 | rs10106152 | T | C | -0.090001 | 0.0186 | 1.31E-06 |  | 185,353 | 191,924 | -0.00190379 | 0.00660664 | 0.773222 |
| 5 | rs10120508 | A | G | -0.0727 | 0.0148 | 9.01E-07 |  | 185,353 | 191,924 | 0.00385601 | 0.00631788 | 0.541642 |
| 6 | rs10492859 | G | A | -0.073297 | 0.016 | 4.63E-06 |  | 185,353 | 191,924 | -0.00912029 | 0.00637678 | 0.15265 |
| 7 | rs10744560 | T | C | 0.083201 | 0.014 | 2.80E-09 |  | 185,353 | 191,924 | -0.00173348 | 0.00601894 | 0.773343 |
| 8 | rs10878840 | A | G | -0.063696 | 0.0138 | 3.92E-06 |  | 185,353 | 191,924 | 0.00511369 | 0.00579108 | 0.37722 |
| 9 | rs10896090 | G | A | -0.089704 | 0.0173 | 2.16E-07 |  | 185,353 | 191,924 | 0.00175159 | 0.00724055 | 0.808847 |
| 10 | rs10994318 | C | G | 0.1409 | 0.0279 | 4.41E-07 |  | 185,353 | 191,924 | -0.00527247 | 0.00763574 | 0.489881 |
| 11 | rs11097326 | A | G | 0.071399 | 0.0149 | 1.65E-06 |  | 185,353 | 191,924 | -0.00699037 | 0.00616754 | 0.257041 |
| 12 | rs111725205 | G | C | 0.089301 | 0.0179 | 6.07E-07 |  | 185,353 | 191,924 | 0.00030326 | 0.00802915 | 0.969871 |
| 13 | rs11557713 | A | G | 0.0718 | 0.0148 | 1.23E-06 |  | 185,353 | 191,924 | 0.00997407 | 0.00632004 | 0.114528 |
| 14 | rs11647445 | G | T | 0.074896 | 0.0142 | 1.33E-07 |  | 185,353 | 191,924 | 0.00249492 | 0.00611433 | 0.683241 |
| 15 | rs11724116 | T | C | -0.104095 | 0.0188 | 3.08E-08 |  | 185,353 | 191,924 | -0.00415997 | 0.00809029 | 0.607118 |
| 16 | rs12135727 | T | C | -0.076104 | 0.0165 | 3.98E-06 |  | 185,353 | 191,924 | -0.0153768 | 0.00682492 | 0.0242566 |
| 17 | rs12287648 | A | G | -0.089695 | 0.0182 | 8.29E-07 |  | 185,353 | 191,924 | 0.00120967 | 0.00646171 | 0.851499 |
| 18 | rs12474837 | C | A | 0.138802 | 0.0287 | 1.32E-06 |  | 185,353 | 191,924 | 0.0255463 | 0.0154342 | 0.0978904 |
| 19 | rs12538191 | A | G | -0.095795 | 0.0182 | 1.41E-07 |  | 185,353 | 191,924 | -0.00415079 | 0.00819292 | 0.612414 |
| 20 | rs12563424 | C | T | 0.069704 | 0.0139 | 5.31E-07 |  | 185,353 | 191,924 | -0.0051061 | 0.00561172 | 0.362875 |
| 21 | rs12621381 | C | A | 0.067797 | 0.0139 | 1.07E-06 |  | 185,353 | 191,924 | 0.00797138 | 0.00565105 | 0.158362 |
| 22 | rs12639551 | C | G | 0.1028 | 0.0225 | 4.90E-06 |  | 185,353 | 191,924 | 0.00871467 | 0.00985113 | 0.376353 |
| 23 | rs12703284 | G | C | 0.086604 | 0.0163 | 1.08E-07 |  | 185,353 | 191,924 | 0.00838749 | 0.00738842 | 0.256283 |
| 24 | rs13003404 | C | T | 0.0727 | 0.0137 | 1.12E-07 |  | 185,353 | 191,924 | -0.0133343 | 0.00568145 | 0.0189256 |
| 25 | rs13231398 | C | G | -0.1207 | 0.0219 | 3.56E-08 |  | 185,353 | 191,924 | -0.00233146 | 0.0108812 | 0.83034 |
| 26 | rs138312 | T | C | 0.074096 | 0.0134 | 3.21E-08 |  | 185,353 | 191,924 | 0.00630675 | 0.00566898 | 0.265922 |
| 27 | rs143577122 | T | C | -0.184199 | 0.0395 | 3.11E-06 |  | 185,353 | 191,924 | -0.021434 | 0.0220584 | 0.331201 |
| 28 | rs16842765 | A | G | -0.066599 | 0.0145 | 4.37E-06 |  | 185,353 | 191,924 | 0.0101006 | 0.00607695 | 0.0964895 |
| 29 | rs17183814 | A | G | -0.140896 | 0.0268 | 1.46E-07 |  | 185,353 | 191,924 | 0.0114983 | 0.0129795 | 0.375681 |
| 30 | rs17566118 | T | C | -0.072399 | 0.0152 | 1.91E-06 |  | 185,353 | 191,924 | 0.00881596 | 0.00665654 | 0.185369 |
| 31 | rs1819204 | A | G | 0.080704 | 0.0162 | 6.30E-07 |  | 185,353 | 191,924 | -0.00998906 | 0.00625217 | 0.110111 |
| 32 | rs185308 | T | C | 0.068499 | 0.0138 | 6.92E-07 |  | 185,353 | 191,924 | -0.0098994 | 0.0058369 | 0.0898856 |
| 33 | rs189574365 | A | C | 0.206803 | 0.0435 | 1.99E-06 |  | 185,353 | 191,924 | 0.00189971 | 0.0192779 | 0.921501 |
| 34 | rs2068756 | A | T | 0.0908 | 0.0167 | 5.41E-08 |  | 185,353 | 191,924 | 0.00863341 | 0.00715271 | 0.227428 |
| 35 | rs2143943 | T | G | -0.065702 | 0.0138 | 1.93E-06 |  | 185,353 | 191,924 | 0.0076063 | 0.00570511 | 0.182452 |
| 36 | rs2597374 | T | A | -0.0692 | 0.0147 | 2.51E-06 |  | 185,353 | 191,924 | 0.00398477 | 0.0064044 | 0.533815 |
| 37 | rs2635253 | T | C | 0.067902 | 0.0135 | 4.91E-07 |  | 185,353 | 191,924 | 0.000425342 | 0.0056941 | 0.940455 |
| 38 | rs28565152 | A | G | 0.080298 | 0.0158 | 3.73E-07 |  | 185,353 | 191,924 | -0.00958362 | 0.00668343 | 0.15159 |
| 39 | rs2877947 | C | T | 0.091797 | 0.0191 | 1.54E-06 |  | 185,353 | 191,924 | -0.00761984 | 0.00857108 | 0.373994 |
| 40 | rs2921552 | A | T | 0.074402 | 0.016 | 3.32E-06 |  | 185,353 | 191,924 | -0.00355545 | 0.00610402 | 0.560245 |
| 41 | rs325380 | C | A | 0.064805 | 0.0137 | 2.24E-06 |  | 185,353 | 191,924 | 0.00747 | 0.00569065 | 0.189291 |
| 42 | rs329319 | G | A | -0.078802 | 0.0139 | 1.43E-08 |  | 185,353 | 191,924 | -0.00794484 | 0.00573414 | 0.16589 |
| 43 | rs34520165 | T | C | 0.0743 | 0.0156 | 1.91E-06 |  | 185,353 | 191,924 | 0.00414971 | 0.0057915 | 0.473672 |
| 44 | rs34568676 | A | G | 0.074402 | 0.0159 | 2.88E-06 |  | 185,353 | 191,924 | -0.00750453 | 0.00685488 | 0.273616 |
| 45 | rs35955717 | C | T | -0.094501 | 0.0198 | 1.82E-06 |  | 185,353 | 191,924 | 0.00441428 | 0.00842073 | 0.600128 |
| 46 | rs36034627 | T | G | -0.078697 | 0.0171 | 4.18E-06 |  | 185,353 | 191,924 | 0.0100579 | 0.00875674 | 0.250726 |
| 47 | rs3911862 | G | A | -0.0635 | 0.0134 | 2.15E-06 |  | 185,353 | 191,924 | -0.0052293 | 0.00564563 | 0.354313 |
| 48 | rs4595478 | C | T | -0.092798 | 0.0178 | 1.85E-07 |  | 185,353 | 191,924 | 0.000527109 | 0.00807607 | 0.947961 |
| 49 | rs4799092 | G | C | 0.067904 | 0.014 | 1.23E-06 |  | 185,353 | 191,924 | 0.00431504 | 0.00616856 | 0.484226 |
| 50 | rs55648125 | G | A | 0.117096 | 0.0215 | 5.14E-08 |  | 185,353 | 191,924 | -0.019444 | 0.00849092 | 0.0220227 |
| 51 | rs56012312 | T | C | -0.066097 | 0.0142 | 3.24E-06 |  | 185,353 | 191,924 | -9.02E-05 | 0.00561045 | 0.987179 |
| 52 | rs570098 | G | T | 0.065296 | 0.0138 | 2.23E-06 |  | 185,353 | 191,924 | -0.0116288 | 0.00558634 | 0.037374 |
| 53 | rs57681866 | A | G | -0.161402 | 0.0296 | 4.96E-08 |  | 185,353 | 191,924 | 0.00315669 | 0.0138204 | 0.819329 |
| 54 | rs6090435 | A | G | -0.0755 | 0.015 | 4.82E-07 |  | 185,353 | 191,924 | 0.00931685 | 0.00561061 | 0.0967988 |
| 55 | rs6102677 | C | T | 0.067904 | 0.0148 | 4.47E-06 |  | 185,353 | 191,924 | 0.00319108 | 0.00636229 | 0.615976 |
| 56 | rs61088439 | A | T | 0.0966 | 0.0183 | 1.30E-07 |  | 185,353 | 191,924 | -0.0052608 | 0.00659778 | 0.425242 |
| 57 | rs62002181 | C | G | 0.095201 | 0.0191 | 6.22E-07 |  | 185,353 | 191,924 | 0.00614783 | 0.0076212 | 0.419854 |
| 58 | rs62433108 | G | T | 0.0683 | 0.0148 | 3.93E-06 |  | 185,353 | 191,924 | -0.011586 | 0.00606687 | 0.0561694 |
| 59 | rs66506713 | T | C | 0.0718 | 0.0157 | 4.80E-06 |  | 185,353 | 191,924 | -0.0094999 | 0.0056298 | 0.0915208 |
| 60 | rs6767302 | G | A | -0.0703 | 0.0135 | 1.91E-07 |  | 185,353 | 191,924 | 0.0116914 | 0.00558069 | 0.0361735 |
| 61 | rs6782817 | A | C | -0.084905 | 0.0183 | 3.49E-06 |  | 185,353 | 191,924 | -0.00550808 | 0.00678162 | 0.416673 |
| 62 | rs6829845 | G | A | -0.072897 | 0.0142 | 2.84E-07 |  | 185,353 | 191,924 | -0.0084963 | 0.00564978 | 0.132626 |
| 63 | rs72927105 | A | T | 0.116004 | 0.0246 | 2.41E-06 |  | 185,353 | 191,924 | -0.00694925 | 0.0112128 | 0.535417 |
| 64 | rs73406518 | T | C | 0.118503 | 0.0235 | 4.59E-07 |  | 185,353 | 191,924 | -0.00578987 | 0.00951705 | 0.542943 |
| 65 | rs73496688 | A | T | 0.108702 | 0.019 | 1.06E-08 |  | 185,353 | 191,924 | 0.00348388 | 0.00979954 | 0.722204 |
| 66 | rs735931 | G | A | -0.066901 | 0.0138 | 1.25E-06 |  | 185,353 | 191,924 | -0.00377802 | 0.00560882 | 0.500576 |
| 67 | rs7613933 | A | G | -0.066599 | 0.0137 | 1.17E-06 |  | 185,353 | 191,924 | -0.00511847 | 0.00563264 | 0.3635 |
| 68 | rs7708829 | G | A | -0.064701 | 0.0135 | 1.65E-06 |  | 185,353 | 191,924 | -0.00224761 | 0.00565262 | 0.690909 |
| 69 | rs77516904 | G | A | 0.170006 | 0.0355 | 1.68E-06 |  | 185,353 | 191,924 | 0.0188066 | 0.0120697 | 0.119194 |
| 70 | rs78781559 | A | G | 0.117499 | 0.0254 | 3.73E-06 |  | 185,353 | 191,924 | 0.00603882 | 0.010459 | 0.563682 |
| 71 | rs7915021 | T | C | -0.099202 | 0.02 | 7.05E-07 |  | 185,353 | 191,924 | -0.0171749 | 0.00817899 | 0.0357396 |
| 72 | rs7916271 | T | C | -0.070401 | 0.0141 | 5.95E-07 |  | 185,353 | 191,924 | 0.0045362 | 0.00577062 | 0.431817 |
| 73 | rs7969091 | G | A | 0.0692 | 0.0135 | 2.96E-07 |  | 185,353 | 191,924 | -0.00209169 | 0.00558734 | 0.708135 |
| 74 | rs80148877 | C | T | 0.150497 | 0.0309 | 1.11E-06 |  | 185,353 | 191,924 | -0.0154061 | 0.0100593 | 0.12564 |
| 75 | rs8067817 | T | C | 0.068397 | 0.0136 | 4.93E-07 |  | 185,353 | 191,924 | -0.00122509 | 0.00561952 | 0.827425 |
| 76 | rs814197 | G | T | 0.070498 | 0.0134 | 1.43E-07 |  | 185,353 | 191,924 | -0.0052421 | 0.00567569 | 0.355692 |
| 77 | rs884301 | T | C | 0.080298 | 0.0138 | 5.93E-09 |  | 185,353 | 191,924 | 0.00806419 | 0.0057241 | 0.158891 |
| 78 | rs9371601 | T | G | 0.066499 | 0.0139 | 1.72E-06 |  | 185,353 | 191,924 | 0.000542628 | 0.00590978 | 0.926842 |
| 79 | rs9834970 | C | T | 0.101003 | 0.0134 | 4.79E-14 |  | 185,353 | 191,924 | -0.007166 | 0.00570601 | 0.209164 |

SNP, single nucleotide polymorphism; EA, effect allele; OA, other allele; SE, standard error; BD, bipolar disorder; CVD, cardiovascular diseases.

**Supplementary Table S2.6** SNPs from GWAS on Depression and CVD

|  |  | | | **Exposure (Depression)** | | |  | **Outcome (CVD)** | | | | |
| --- | --- | --- | --- | --- | --- | --- | --- | --- | --- | --- | --- | --- |
|  | **SNP** | **EA** | **OA** | **β** | **SE** | ***p* value** |  | **Case** | **Control** | **β** | **SE** | ***p* value** |
| 1 | rs10088768 | C | G | 0.0425151 | 0.0093036 | 4.88E-06 |  | 185,353 | 191,924 | 0.00648835 | 0.00699879 | 0.353891 |
| 2 | rs10164593 | T | C | -0.0501523 | 0.0107868 | 3.33E-06 |  | 185,353 | 191,924 | 0.00567859 | 0.0079953 | 0.477555 |
| 3 | rs1027190 | G | T | -0.0459014 | 0.00834093 | 3.73E-08 |  | 185,353 | 191,924 | -0.00957134 | 0.00626297 | 0.126452 |
| 4 | rs10507576 | A | G | 0.0949104 | 0.0188169 | 4.56E-07 |  | 185,353 | 191,924 | -0.0193326 | 0.0142912 | 0.17613 |
| 5 | rs10759925 | C | T | -0.0375299 | 0.00786224 | 1.81E-06 |  | 185,353 | 191,924 | -0.00462336 | 0.00585766 | 0.429946 |
| 6 | rs10990411 | C | T | -0.0543872 | 0.0113176 | 1.54E-06 |  | 185,353 | 191,924 | -0.0231221 | 0.00839127 | 0.0058603 |
| 7 | rs11109659 | C | A | 0.0601382 | 0.0130426 | 4.01E-06 |  | 185,353 | 191,924 | 0.007387 | 0.00985158 | 0.453358 |
| 8 | rs11159917 | T | C | 0.0402987 | 0.00866968 | 3.35E-06 |  | 185,353 | 191,924 | -0.00362886 | 0.00651982 | 0.577809 |
| 9 | rs113661867 | T | C | 0.0965125 | 0.0184234 | 1.62E-07 |  | 185,353 | 191,924 | 0.0400343 | 0.0141935 | 0.00479325 |
| 10 | rs113823870 | A | G | -0.0785054 | 0.0151251 | 2.10E-07 |  | 185,353 | 191,924 | 0.00858625 | 0.0111971 | 0.443184 |
| 11 | rs113909596 | A | G | 0.105733 | 0.0214016 | 7.80E-07 |  | 185,353 | 191,924 | -0.0165109 | 0.0164505 | 0.315538 |
| 12 | rs11743963 | T | C | -0.0354189 | 0.00751584 | 2.45E-06 |  | 185,353 | 191,924 | 0.00105644 | 0.00561607 | 0.850791 |
| 13 | rs11756123 | T | A | -0.0539258 | 0.00775723 | 3.61E-12 |  | 185,353 | 191,924 | -0.0105035 | 0.00582483 | 0.071351 |
| 14 | rs117763266 | T | C | -0.101108 | 0.0221313 | 4.91E-06 |  | 185,353 | 191,924 | -0.0201645 | 0.016252 | 0.214703 |
| 15 | rs12479064 | T | C | 0.0555961 | 0.0099119 | 2.03E-08 |  | 185,353 | 191,924 | 0.012464 | 0.00749002 | 0.0960948 |
| 16 | rs12602745 | C | T | -0.0412079 | 0.00901495 | 4.85E-06 |  | 185,353 | 191,924 | 0.00617349 | 0.00670757 | 0.357377 |
| 17 | rs12712502 | T | C | -0.0373703 | 0.0074975 | 6.22E-07 |  | 185,353 | 191,924 | -0.0143702 | 0.0056003 | 0.0102889 |
| 18 | rs12763632 | G | A | 0.0415112 | 0.00906568 | 4.67E-06 |  | 185,353 | 191,924 | 0.0084151 | 0.00681223 | 0.216722 |
| 19 | rs12804093 | C | T | 0.038152 | 0.00793574 | 1.53E-06 |  | 185,353 | 191,924 | 0.000722165 | 0.00594687 | 0.903346 |
| 20 | rs13094224 | G | A | 0.0430883 | 0.00873661 | 8.14E-07 |  | 185,353 | 191,924 | 0.0199276 | 0.00655467 | 0.00236412 |
| 21 | rs13120249 | A | G | 0.0628108 | 0.0137475 | 4.90E-06 |  | 185,353 | 191,924 | 0.0259231 | 0.0103613 | 0.0123518 |
| 22 | rs13249798 | G | C | -0.0602644 | 0.0125246 | 1.50E-06 |  | 185,353 | 191,924 | -0.0184316 | 0.00928301 | 0.0470858 |
| 23 | rs140005264 | T | G | 0.0806647 | 0.0162097 | 6.48E-07 |  | 185,353 | 191,924 | 0.0251247 | 0.0124067 | 0.0428568 |
| 24 | rs142330459 | A | G | 0.120758 | 0.0258578 | 3.01E-06 |  | 185,353 | 191,924 | 0.0215691 | 0.0199143 | 0.278765 |
| 25 | rs144644837 | A | G | 0.0689059 | 0.0137101 | 5.01E-07 |  | 185,353 | 191,924 | 0.0304732 | 0.0103981 | 0.00338244 |
| 26 | rs147831713 | C | A | 0.040316 | 0.00783273 | 2.65E-07 |  | 185,353 | 191,924 | -0.00149982 | 0.00587173 | 0.798391 |
| 27 | rs1479551 | G | A | -0.0368752 | 0.00752535 | 9.58E-07 |  | 185,353 | 191,924 | 0.00346186 | 0.00562134 | 0.537998 |
| 28 | rs17117590 | G | A | 0.0998261 | 0.0209482 | 1.88E-06 |  | 185,353 | 191,924 | 0.00266696 | 0.0160379 | 0.867928 |
| 29 | rs190517796 | T | C | 0.0976498 | 0.019128 | 3.31E-07 |  | 185,353 | 191,924 | 0.0062809 | 0.014826 | 0.671828 |
| 30 | rs192578494 | C | A | 0.392974 | 0.085095 | 3.87E-06 |  | 185,353 | 191,924 | -0.03739 | 0.0691422 | 0.588666 |
| 31 | rs1995514 | A | G | -0.0416291 | 0.00769068 | 6.20E-08 |  | 185,353 | 191,924 | -0.00620488 | 0.00572661 | 0.278579 |
| 32 | rs215896 | C | T | 0.0519425 | 0.00863932 | 1.83E-09 |  | 185,353 | 191,924 | 0.00760996 | 0.00650512 | 0.242065 |
| 33 | rs2187987 | T | G | 0.0407482 | 0.00821121 | 6.96E-07 |  | 185,353 | 191,924 | 0.012692 | 0.00616121 | 0.0394003 |
| 34 | rs259446 | C | T | -0.0399854 | 0.00798908 | 5.59E-07 |  | 185,353 | 191,924 | -0.00207133 | 0.0060008 | 0.729963 |
| 35 | rs3130160 | G | A | 0.0374324 | 0.00812027 | 4.03E-06 |  | 185,353 | 191,924 | 0.0043087 | 0.00608984 | 0.479241 |
| 36 | rs34115597 | G | T | -0.0373173 | 0.00813691 | 4.51E-06 |  | 185,353 | 191,924 | 0.000399741 | 0.00606544 | 0.947454 |
| 37 | rs34852515 | C | T | -0.0380097 | 0.00819357 | 3.50E-06 |  | 185,353 | 191,924 | -0.00788729 | 0.0061013 | 0.196107 |
| 38 | rs35487006 | T | G | -0.0360396 | 0.00765596 | 2.51E-06 |  | 185,353 | 191,924 | -0.00620833 | 0.00571018 | 0.276932 |
| 39 | rs35792797 | T | A | -0.0424795 | 0.00841312 | 4.44E-07 |  | 185,353 | 191,924 | 0.00463798 | 0.00626416 | 0.459057 |
| 40 | rs36090777 | C | G | -0.0597606 | 0.0122893 | 1.16E-06 |  | 185,353 | 191,924 | -0.0163424 | 0.00906347 | 0.0713707 |
| 41 | rs3773087 | C | T | -0.059413 | 0.0108625 | 4.51E-08 |  | 185,353 | 191,924 | -0.0182452 | 0.00803661 | 0.0231916 |
| 42 | rs3792736 | T | A | -0.0362726 | 0.00761308 | 1.89E-06 |  | 185,353 | 191,924 | -0.00834105 | 0.00569054 | 0.14271 |
| 43 | rs4348025 | G | A | -0.0403294 | 0.00751699 | 8.09E-08 |  | 185,353 | 191,924 | -0.018214 | 0.00562212 | 0.00119658 |
| 44 | rs4732435 | G | A | 0.0397918 | 0.00786268 | 4.17E-07 |  | 185,353 | 191,924 | 0.0100943 | 0.00585243 | 0.0845649 |
| 45 | rs4882348 | T | C | -0.0381598 | 0.00750864 | 3.73E-07 |  | 185,353 | 191,924 | 0.00291957 | 0.00561281 | 0.602951 |
| 46 | rs530758485 | A | G | 0.081333 | 0.011856 | 6.88E-12 |  | 185,353 | 191,924 | 0.00652198 | 0.00908808 | 0.472978 |
| 47 | rs534804 | A | G | 0.0447515 | 0.00922321 | 1.22E-06 |  | 185,353 | 191,924 | 0.00227413 | 0.00691638 | 0.742304 |
| 48 | rs57268239 | A | C | 0.0510729 | 0.00926038 | 3.48E-08 |  | 185,353 | 191,924 | 0.00550408 | 0.00697903 | 0.430311 |
| 49 | rs57852066 | A | G | 0.0941695 | 0.0168294 | 2.20E-08 |  | 185,353 | 191,924 | 0.0145711 | 0.0129586 | 0.260828 |
| 50 | rs587925 | G | A | -0.0402559 | 0.00776064 | 2.13E-07 |  | 185,353 | 191,924 | 0.000824402 | 0.00582896 | 0.887528 |
| 51 | rs6707756 | C | T | -0.0458768 | 0.00830476 | 3.31E-08 |  | 185,353 | 191,924 | -0.0107314 | 0.00617765 | 0.0823626 |
| 52 | rs67090447 | A | G | -0.0499982 | 0.010725 | 3.13E-06 |  | 185,353 | 191,924 | -0.00898797 | 0.00798987 | 0.260623 |
| 53 | rs6876567 | G | A | 0.0508467 | 0.00763157 | 2.69E-11 |  | 185,353 | 191,924 | 0.00255386 | 0.00571605 | 0.655028 |
| 54 | rs6890818 | T | C | 0.0465145 | 0.00952902 | 1.05E-06 |  | 185,353 | 191,924 | 0.00233001 | 0.00719376 | 0.746019 |
| 55 | rs7184244 | A | C | 0.0357005 | 0.00755322 | 2.28E-06 |  | 185,353 | 191,924 | -0.000573275 | 0.00564135 | 0.919058 |
| 56 | rs72481178 | C | T | -0.0880699 | 0.0185334 | 2.01E-06 |  | 185,353 | 191,924 | -0.00849557 | 0.0136405 | 0.533404 |
| 57 | rs72848297 | G | A | 0.0638356 | 0.0132281 | 1.39E-06 |  | 185,353 | 191,924 | -0.00253133 | 0.00998797 | 0.799929 |
| 58 | rs75540846 | T | C | 0.0475048 | 0.0102842 | 3.85E-06 |  | 185,353 | 191,924 | 0.0138046 | 0.00773595 | 0.0743481 |
| 59 | rs7556072 | A | G | -0.0378194 | 0.00757021 | 5.86E-07 |  | 185,353 | 191,924 | -0.00517613 | 0.00565685 | 0.360181 |
| 60 | rs7595691 | G | A | -0.0747038 | 0.0162216 | 4.12E-06 |  | 185,353 | 191,924 | 0.0159249 | 0.0118788 | 0.180044 |
| 61 | rs771124 | C | T | -0.0656875 | 0.0137377 | 1.74E-06 |  | 185,353 | 191,924 | -0.00148024 | 0.0104345 | 0.887191 |
| 62 | rs7842714 | A | T | 0.0365288 | 0.00774148 | 2.37E-06 |  | 185,353 | 191,924 | 0.00441658 | 0.00576278 | 0.44344 |
| 63 | rs927456 | C | T | 0.0365299 | 0.00771541 | 2.19E-06 |  | 185,353 | 191,924 | 0.0184785 | 0.00577612 | 0.00137848 |
| 64 | rs9293745 | A | G | 0.040005 | 0.00838697 | 1.84E-06 |  | 185,353 | 191,924 | 0.00408611 | 0.00623168 | 0.512018 |
| 65 | rs9296100 | T | C | 0.0434263 | 0.00884014 | 9.00E-07 |  | 185,353 | 191,924 | 0.0044115 | 0.00655647 | 0.501044 |
| 66 | rs9324959 | A | G | 0.0448681 | 0.00767423 | 5.02E-09 |  | 185,353 | 191,924 | 0.00877535 | 0.00574427 | 0.126594 |
| 67 | rs940348 | G | A | 0.0477782 | 0.00888347 | 7.52E-08 |  | 185,353 | 191,924 | 0.0188915 | 0.00659871 | 0.00419778 |
| 68 | rs9491216 | G | T | 0.0384761 | 0.00760363 | 4.19E-07 |  | 185,353 | 191,924 | 0.00367317 | 0.00567179 | 0.51723 |
| 69 | rs9570498 | A | C | -0.070633 | 0.0153391 | 4.13E-06 |  | 185,353 | 191,924 | -0.00993826 | 0.0112985 | 0.379071 |
| 70 | rs978164 | A | G | -0.0393219 | 0.00751737 | 1.69E-07 |  | 185,353 | 191,924 | -0.00751438 | 0.00562186 | 0.181342 |
| 71 | rs9890900 | C | T | 0.0630472 | 0.0123265 | 3.14E-07 |  | 185,353 | 191,924 | 0.00364288 | 0.00927462 | 0.694482 |
| 72 | rs9916184 | T | G | 0.0612116 | 0.0114653 | 9.35E-08 |  | 185,353 | 191,924 | 0.0120317 | 0.008599 | 0.161755 |

SNP, single nucleotide polymorphism; EA, effect allele; OA, other allele; SE, standard error; CVD, cardiovascular diseases.

**Supplementary Table S2.7** SNPs from GWAS on OCD and CVD

|  |  | | | **Exposure (OCD)** | | |  | **Outcome (CVD)** | | | | |
| --- | --- | --- | --- | --- | --- | --- | --- | --- | --- | --- | --- | --- |
|  | **SNP** | **EA** | **OA** | **β** | **SE** | ***p* value** |  | **Case** | **Control** | **β** | **SE** | ***p* value** |
| 1 | rs11717238 | G | A | -0.149474 | 0.032662 | 4.73E-06 |  | 185,353 | 191,924 | -0.00114142 | 0.00565756 | 0.840111 |
| 2 | rs118191920 | A | G | -0.389995 | 0.0788855 | 7.66E-07 |  | 185,353 | 191,924 | -0.00075946 | 0.0122396 | 0.950524 |
| 3 | rs12213997 | C | T | 0.198928 | 0.0434961 | 4.80E-06 |  | 185,353 | 191,924 | 0.00381371 | 0.00781101 | 0.625374 |
| 4 | rs13427136 | C | T | 0.273179 | 0.052694 | 2.17E-07 |  | 185,353 | 191,924 | -0.0167417 | 0.00959489 | 0.0810103 |
| 5 | rs17466747 | A | G | 0.175437 | 0.0348176 | 4.69E-07 |  | 185,353 | 191,924 | 0.00517495 | 0.00614397 | 0.399631 |
| 6 | rs315678 | T | C | 0.269177 | 0.0565215 | 1.91E-06 |  | 185,353 | 191,924 | -0.00857109 | 0.00910673 | 0.346612 |
| 7 | rs55888334 | A | G | 0.315626 | 0.0627743 | 4.96E-07 |  | 185,353 | 191,924 | -0.0188879 | 0.0117506 | 0.107967 |
| 8 | rs58466321 | C | T | 0.272631 | 0.0570772 | 1.78E-06 |  | 185,353 | 191,924 | -0.0172644 | 0.0105216 | 0.100828 |
| 9 | rs60562275 | T | C | 0.238119 | 0.0518566 | 4.39E-06 |  | 185,353 | 191,924 | -0.00799191 | 0.00943477 | 0.396956 |
| 10 | rs6105964 | A | G | 0.245975 | 0.0537313 | 4.70E-06 |  | 185,353 | 191,924 | 0.00962551 | 0.00987973 | 0.329923 |
| 11 | rs7207457 | T | C | -0.170155 | 0.0356018 | 1.76E-06 |  | 185,353 | 191,924 | 0.00838979 | 0.00604341 | 0.16506 |
| 12 | rs74924645 | A | G | -0.266287 | 0.0562867 | 2.24E-06 |  | 185,353 | 191,924 | 0.00470563 | 0.00912735 | 0.606167 |
| 13 | rs76110959 | G | A | -0.882499 | 0.192575 | 4.59E-06 |  | 185,353 | 191,924 | 0.0249507 | 0.0255736 | 0.32924 |
| 14 | rs79239243 | A | C | 0.453007 | 0.0979061 | 3.71E-06 |  | 185,353 | 191,924 | -0.0283581 | 0.0195144 | 0.146172 |
| 15 | rs80216287 | T | G | 0.216118 | 0.0452259 | 1.76E-06 |  | 185,353 | 191,924 | 0.0127499 | 0.00819461 | 0.119735 |
| 16 | rs8084719 | G | C | -0.185983 | 0.0370263 | 5.09E-07 |  | 185,353 | 191,924 | -0.00346479 | 0.00623605 | 0.578481 |

SNP, single nucleotide polymorphism; EA, effect allele; OA, other allele; SE, standard error; OCD, obsessive compulsive disorder; CVD, cardiovascular diseases.

**Supplementary Table S2.8** SNPs from GWAS on SCZ and CVD

|  |  | | | **Exposure (SCZ)** | | |  | **Outcome (CVD)** | | | | |
| --- | --- | --- | --- | --- | --- | --- | --- | --- | --- | --- | --- | --- |
|  | **SNP** | **EA** | **OA** | **β** | **SE** | ***p* value** |  | **Case** | **Control** | **β** | **SE** | ***p* value** |
| 1 | rs12652777 | C | T | -0.125906 | 0.0262846 | 1.67E-06 |  | 185,353 | 191,924 | -0.0101872 | 0.00561812 | 0.0697895 |
| 2 | rs144031898 | T | C | -0.358446 | 0.0776489 | 3.91E-06 |  | 185,353 | 191,924 | 0.00561637 | 0.0159 | 0.723916 |
| 3 | rs1641714 | A | G | 0.143081 | 0.0299733 | 1.81E-06 |  | 185,353 | 191,924 | 0.0122065 | 0.00646081 | 0.0588491 |
| 4 | rs2675600 | A | G | -0.180013 | 0.0391568 | 4.28E-06 |  | 185,353 | 191,924 | 0.00221669 | 0.00865015 | 0.797749 |
| 5 | rs58968563 | T | C | -0.276263 | 0.0549836 | 5.05E-07 |  | 185,353 | 191,924 | -0.0121338 | 0.0112894 | 0.282465 |
| 6 | rs59665366 | A | C | 0.156932 | 0.0341188 | 4.23E-06 |  | 185,353 | 191,924 | 0.00107148 | 0.00743129 | 0.885354 |
| 7 | rs62203513 | A | G | -0.162663 | 0.0354067 | 4.35E-06 |  | 185,353 | 191,924 | -0.00361232 | 0.00750073 | 0.630094 |
| 8 | rs6966281 | T | G | 0.13287 | 0.027283 | 1.12E-06 |  | 185,353 | 191,924 | -0.00260707 | 0.00577517 | 0.651681 |
| 9 | rs72827147 | G | T | -0.28163 | 0.0590435 | 1.84E-06 |  | 185,353 | 191,924 | 0.00284877 | 0.0121631 | 0.814819 |
| 10 | rs77012313 | C | A | -0.459696 | 0.0993477 | 3.71E-06 |  | 185,353 | 191,924 | 0.00214893 | 0.019699 | 0.913132 |
| 11 | rs80141503 | A | G | 0.126344 | 0.0276429 | 4.86E-06 |  | 185,353 | 191,924 | -0.00370755 | 0.00585568 | 0.526633 |
| 12 | rs9367849 | G | C | 0.206682 | 0.0414786 | 6.27E-07 |  | 185,353 | 191,924 | -0.0185178 | 0.00898028 | 0.0392031 |

SNP, single nucleotide polymorphism; EA, effect allele; OA, other allele; SE, standard error; SCZ, schizophrenia; CVD, cardiovascular diseases.

**Supplementary Table S3** The results of MR-Egger intercept analysis

| **Exposure** | **Outcome** | **Egger_intercept** | **SE** | ***p* value** |
| --- | --- | --- | --- | --- |
| ADHD | CVD | 0.003876186 | 0.005176437 | 0.460000409 |
| AN | CVD | 0.003512918 | 0.006873364 | 0.621588123 |
| ANX | CVD | 0.004892567 | 0.003406498 | 0.159334314 |
| ASD | CVD | 0.008996973 | 0.005317364 | 0.106169068 |
| BD | CVD | -0.001768327 | 0.003802719 | 0.643232066 |
| Depression | CVD | 0.0019827 | 0.003333627 | 0.553923279 |
| OCD | CVD | 0.009938129 | 0.006172676 | 0.129703167 |
| SCZ | CVD | 0.006089475 | 0.007248315 | 0.420471966 |

CVD, cardiovascular diseases; ADHD, attention deficit hyperactivity disorder; AN, anorexia nervosa; ANX, anxiety disorder; ASD, autism spectrum disorder; BD, bipolar disorder; OCD, obsessive compulsive disorder; SCZ, schizophrenia.

**Supplementary Table S4** The results of heterogeneity analysis

| **Exposure** | **Outcome** | **Method** | **Q** | **Q_df** | **Q_*p* val** |
| --- | --- | --- | --- | --- | --- |
| ADHD | CVD | MR Egger | 36.31741274 | 29 | 0.164499804 |
| ADHD | CVD | Inverse variance weighted | 37.0196184 | 30 | 0.176573291 |
| AN | CVD | MR Egger | 8.223554101 | 9 | 0.511783964 |
| AN | CVD | Inverse variance weighted | 8.484768658 | 10 | 0.581595371 |
| ANX | CVD | MR Egger | 30.19689712 | 37 | 0.778291944 |
| ANX | CVD | Inverse variance weighted | 32.25969808 | 38 | 0.73148516 |
| ASD | CVD | MR Egger | 27.98530768 | 20 | 0.109747624 |
| ASD | CVD | Inverse variance weighted | 31.99120724 | 21 | 0.058670326 |
| BD | CVD | MR Egger | 101.8638952 | 77 | 0.030489098 |
| BD | CVD | Inverse variance weighted | 102.1499611 | 78 | 0.03469354 |
| Depression | CVD | MR Egger | 89.62652783 | 70 | 0.057034779 |
| Depression | CVD | Inverse variance weighted | 90.07944458 | 71 | 0.062822083 |
| OCD | CVD | MR Egger | 14.54713079 | 14 | 0.409794509 |
| OCD | CVD | Inverse variance weighted | 17.24059715 | 15 | 0.304686153 |
| SCZ | CVD | MR Egger | 12.36300794 | 10 | 0.261496 |
| SCZ | CVD | Inverse variance weighted | 13.23559697 | 11 | 0.278206273 |

CVD, cardiovascular diseases; ADHD, attention deficit hyperactivity disorder; AN, anorexia nervosa; ANX, anxiety disorder; ASD, autism spectrum disorder; BD, bipolar disorder; OCD, obsessive compulsive disorder; SCZ, schizophrenia.
