# Supplementary figures and images for: A Mendelian randomization study of the effect of mental disorders on cardiovascular disease
Source: Front Cardiovasc Med. 2024 Jun 3;11:1329463. doi: 10.3389/fcvm.2024.1329463 (PMC11180800; doi:10.3389/fcvm.2024.1329463)

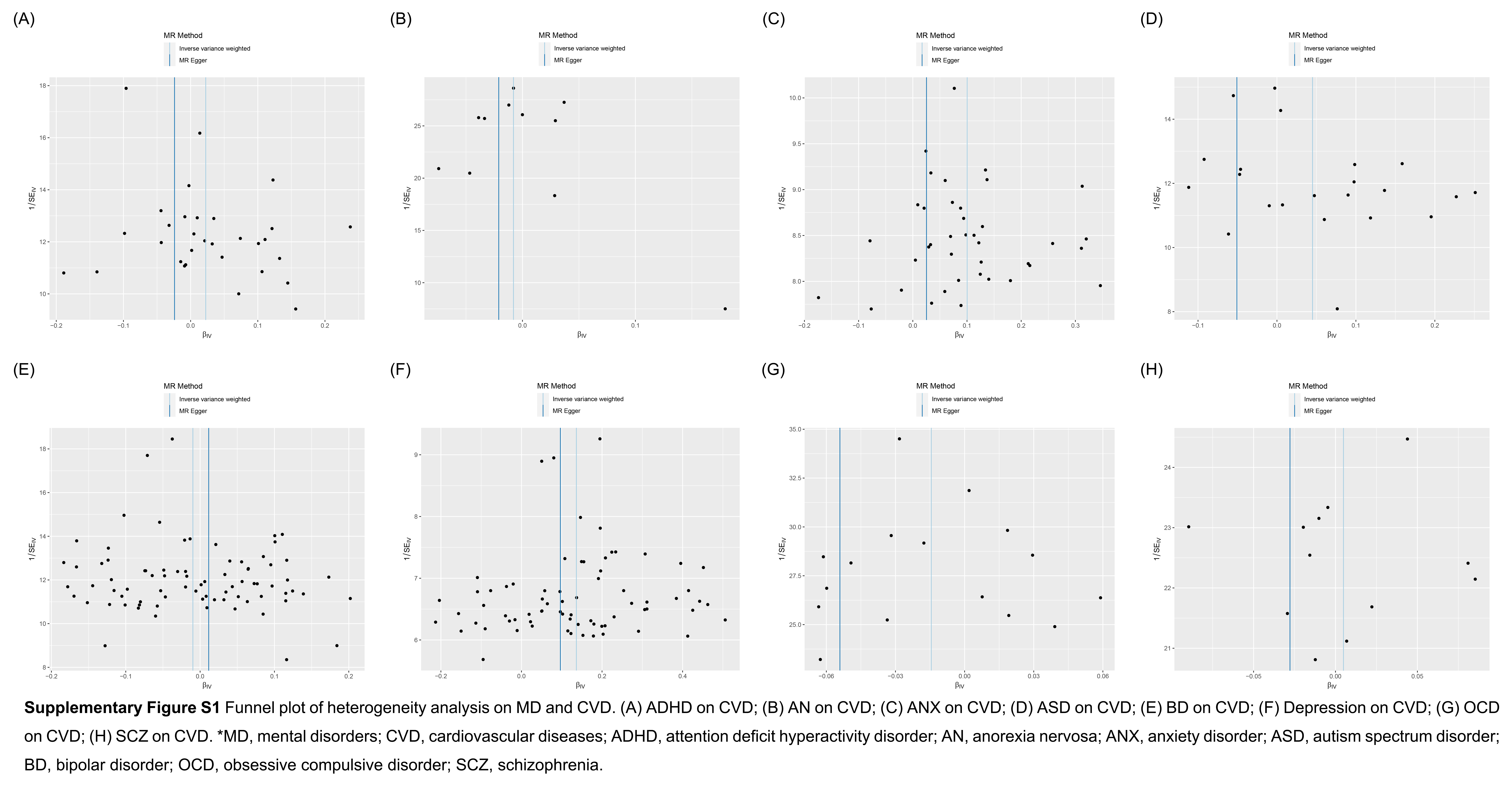

Supplement: Supplementary file 2 [file Image1.jpeg]

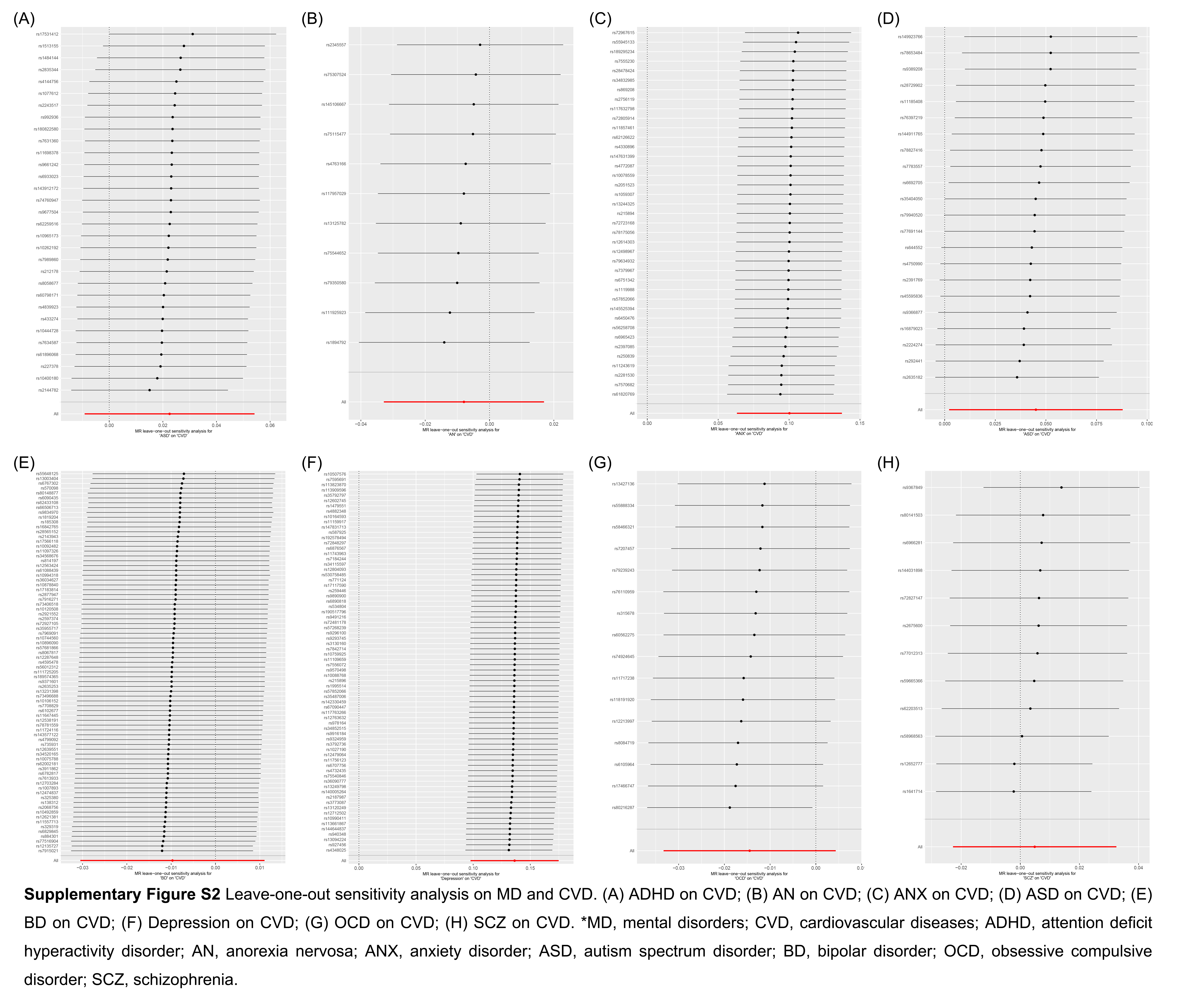

Supplement: Supplementary file 3 [file Image2.jpeg]
